# Long-Standing Activity with Characteristic Genomic Insertion Signatures in Reptilian Bov-B LINEs and Associated Sauria SINEs

**DOI:** 10.3390/biology15120927

**Published:** 2026-06-13

**Authors:** Yoshiki Nakatsuka, Kazuhiko Ohshima

**Affiliations:** Graduate School of Bioscience, Nagahama Institute of Bio-Science and Technology, Nagahama 526-0829, Japan

**Keywords:** Bov-B, RTE clade, long interspersed nuclear element (LINE), short interspersed nuclear element (SINE), target site duplication, horizontal transfer

## Abstract

Although long interspersed nuclear elements (LINEs) and short interspersed nuclear elements (SINEs) are typically passed down to descendants as part of the genome, the Bov-B LINE was likely horizontally transferred from a snake to the ancestor of ruminants. Plant RTE-clade LINEs and their associated SINEs possess a genomic insertion signature different from that of mammalian L1 LINEs. However, the reason for the increased frequency of horizontal transfer in RTE-clade LINEs such as Bov-B relative to that in L1-clade LINEs has not yet been clarified. In this study, we identified family members of the reptilian Bov-B LINE and associated Sauria SINE across various squamate species to determine the amplification timing of the LINE. The findings revealed that the LINE may be over 180 million years old. Moreover, profiling of target site duplications showed that a characteristic genomic insertion signature of the LINE and SINE closely resembled the signature of the plant RTE-clade LINEs. We conducted phylogenetic analyses of RTE-clade LINEs with characteristic genomic insertion signatures and estimated their divergence times. The findings suggest an ancient origin (over 411 MYA) of the retrotranspositional mechanism underlying this signature; however, a complex evolutionary trajectory of LINEs across species warrants further investigation.

## Simple Summary

Long interspersed nuclear elements (LINEs) and short interspersed nuclear elements (SINEs) are retrotransposons that constitute a large proportion of the genomes of multiple animal and plant species. These elements are typically inherited from ancestors and passed down to descendants as part of the genome. However, Bov-B LINE was likely transferred horizontally to a ruminant ancestor from a snake. We demonstrated the long-standing activity of the reptilian Bov-B LINE and revealed a characteristic genomic insertion signature that closely resembles the plant RTE-clade LINE signature in the Bov-B LINE and associated SINE. Although a complex evolutionary trajectory of LINEs across species is plausible, we suggest an ancient origin (over 411 MYA) for the retrotranspositional mechanism underlying this signature. The discovery of novel insertion signatures in distinct clades of LINEs and associated SINEs could contribute to a better understanding of the evolutionary impact of these elements.

## 1. Introduction

Long interspersed nuclear elements (LINEs) are typically inherited from evolutionary ancestors and passed down to descendants as part of the species genome [1,2]. However, recent research has revealed an increasing number of well-supported cases of horizontal transfer (HT) of LINEs [3,4,5,6,7,8,9]. One notable example is the bovine Bov-B LINE family, which was likely introduced from a snake into the ancestor of ruminants via HT [10,11,12]. Phylogenetic analyses of reverse transcriptases (RTs) have revealed that Bov-B LINEs belong to the RTE clade [1,13,14,15]. RTE-clade LINEs have been identified in the genomes of a wide variety of, but not all of, eukaryotes such as mammals, non-mammalian vertebrates, lancelet, insects, nematodes, planarian, cnidarians and flowering plants [15].

Surprisingly, RTE-clade LINEs exhibited frequent HT. Walsh et al. [3] demonstrated that the HT of Bov-B LINEs was more widespread than previously believed, with two plausible arthropod vectors, specifically reptile ticks, playing a role. Our research group [9] also discovered a unique HT pattern of the Bov-B LINE in vertebrates, suggesting its transfer from predators (snakes) to their prey (frogs). Bov-B HT between predators and prey is prevalent in Madagascar [9]. Additionally, Suh et al. [4] observed that the genomes of nematodes and seven tropical bird lineages exclusively shared a novel RTE-clade LINE called AviRTE that resulted from HT. Furthermore, Gao et al. [16] conducted phylogenetic and evolutionary analyses of RTEs from both animals and plants and reported that an angiosperm RTE-clade LINE likely underwent HT from ancient aphids or ancestral arthropods to angiosperms. However, the reasons why RTE-clade LINEs undergo HT more frequently than L1-clade LINEs remain poorly understood [6,7].

**Figure 1:**
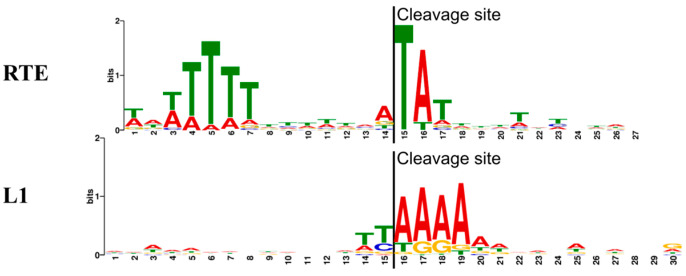
Insertion signatures of the RTE-associated SINE and L1-clade LINE. The first DNA strand cleavage sites that correspond to a phosphodiester bond between the first nucleotide of a 5′ direct repeat and the adjacent 5′ flanking nucleotide on the complementary strand are indicated by a vertical line. (**Upper**) A thymine stretch appears approximately ten nucleotides upstream of the first nucleotide (thymine) of the TSDs in the RTE-associated SINE from soybean [29]. (**Lower**) A typical consensus 5′-TT|AAAA-3′ appears beside the first nucleotide (adenine) of the TSDs in the L1-clade LINE from *Bos taurus* (L1-BT; this study). MEME result for L1-BT with an interval of ≥7999 bases (361 TSDs). The vertical axis represents the information content of the site.

LINEs are inserted into the genome through a mechanism known as target DNA-primed reverse transcription [17,18,19,20]. The LINE insertion site is primarily determined by the DNA cleavage specificity of the endonuclease (EN) domain of the LINE-encoded open reading frame 2 (ORF2) protein [17,21,22,23]. LINEs belonging to more than 20 clades (e.g., L1 and RTE) encode apurinic/apyrimidinic EN-like ENs. Most of them are inserted at multiple loci within the host genome and may exhibit weak target site preference [24], while only two clades, Tx1 and R1, contain site-specific LINEs [25]. For example, human L1 preferentially inserts at sites with the sequence 5′-TT|AAAA-3′, where the vertical bar (|) indicates the site of insertion, and the EN of L1 cleaves the TpA bond on the complementary strand [20,21,26,27] (Figure 1). Target DNA-primed reverse transcription often results in the duplication of a short stretch of nucleotides, typically no more than 20 bp, due to integration at staggered chromosomal breaks. Consequently, each newly inserted LINE is typically flanked by short direct repeats, known as target site duplication (TSD) [27]. Analysis of TSDs has primarily focused on mammalian L1 LINEs [28,29].

Short interspersed nuclear elements (SINEs) are non-autonomous retroposons [30,31,32,33]. Their 5′-end sequences are derived from tRNA [34], 5S rRNA [35], or 7SL RNA [36], while their 3′-end sequences originate from corresponding LINEs [37], except for the L1 LINEs [38,39,40,41]. The analysis of TSDs in SINEs [42] has provided valuable insights into the enzymatic source of SINE retrotransposition [29,43,44,45,46,47,48]. Sauria SINEs represent a SINE family whose members are widely distributed among the genomes of lizards and snakes, and they share a part of their 3′-end sequence with Bov-B LINEs [49]. Therefore, these SINEs likely use the enzymatic machinery of Bov-B LINEs for retrotransposition [49].

In a previous study, we identified a novel insertion signature shared by plant RTE-clade LINEs and related SINEs [29]. These angiosperm SINEs exhibited a novel and remarkable TSD pattern, where a thymine stretch appeared approximately ten nucleotides (one helical pitch) upstream of the first nucleotide of the TSDs (Figure 1). Such a split signature of TSDs has not been previously reported in plants. We observed this pattern in both SINEs and LINEs in the genome of leguminous plants, where the SINEs share the 3′-end sequence with the RTE-clade LINEs. Their TSDs began with thymine—the 3′-end nucleotide next to thymine was typically adenine—and the 3′-ends of the SINEs and LINEs terminated at T_n_ and (GTT)_n_, respectively [29]. We also demonstrated that a horse SINE family exhibits a similar pattern, with a thymine stretch plus TA; however, the TSDs started with adenine while SINE ended with (CAA)_n_. The TSDs of the corresponding RTE-clade LINE tended to start with adenine while the LINE ended with (CAA)_n_ [29]. Gilbert et al. also found a similar TSD pattern in an elephant LINE family from the RTE clade [44].

Based on these observations, we proposed a mechanism underlying these genomic signatures. We hypothesized that the RTE-encoded ORF2 protein would preferentially bind to DNA regions containing thymine stretches, allowing it to cleave a phosphodiester bond downstream of the stretch. Furthermore, microsatellite-like repeats in the RNA template may influence the identification of EN cleavage sites and/or efficiency of reverse transcription initiation [29]. Sauria SINEs in the green anole exhibited the same pattern in terms of the thymine stretch plus TA. Their TSD started with thymine while SINE ended with (ACCTTT)_n_. However, the TSD of the corresponding Bov-B LINE tended to start with adenine while the LINE ended with (CGA)_n_ [29].

In this study, we investigated the genomic insertion patterns of Bov-B LINEs and associated Sauria SINEs across various squamate species, the only reptilian group harboring Bov-B, to elucidate the insertion mechanism of the reptilian Bov-B. We successfully clarified the insertion signatures of the LINE and SINE in the same species and observed long-term activity of the LINE with the characteristic insertion signature.

## 2. Materials and Methods

### 2.1. Nucleotide Sequences

Whole-genome sequences were obtained from Ensembl (including 9 squamates and 6 representative ruminants from the 15 species assembled at chromosomal level) [50] and Khedkar et al. (*Indotyphlops braminus*) [51] (Table S1). Consensus sequences of LINEs and SINEs used as queries for initial basic local alignment search tool (BLAST) searches were retrieved from Repbase [52] and Piskurek et al. [49], respectively (Table S2). The initial survey results for the Bov-B and Sauria SINE in the genomes of reptiles and birds with these sequences as the query are presented in Table S3. The query sequences used for subsequent BLAST searches were extracted from the genomes of respective species (Section 2.2; Table S4).

### 2.2. Extraction of Copy Sequences and Search for TSD

Using the consensus sequences as queries (Table S2), the first blastn search (BLAST+ 2.9.0 [53]) was performed against the whole-genome sequences of 16 or 9 species (for LINEs or SINEs, respectively), with default settings. Based on the search results, the copy sequence (family member) with the highest identity and sequence length closest to the query was selected for each species. Using this sequence as another query (Table S4), the second blastn search was performed against each genome. The resulting copy sequences plus 200 bases of their 5′ and 3′ flanking sequences were extracted from the genomic sequence using the blastdbcmd (BLAST+ 2.9.0 [53]).

Within the extracted sequences, we scanned for TSDs with a Python 3 script (Figure S1) using the following criteria: (1) TSD length is between 10 and 49 bases inclusive; (2) the 5′ and 3′ TSD sequences are perfectly matched; (3) the 5′ and 3′ TSD sequences are separated by at least 99 bases [29]. Due to the long nucleotide sequences of LINEs, direct repeats unrelated to TSDs may exist within the detected sequences. To address this, we examined the presence of direct repeats in the query sequences of the first BLAST searches using the Python script mentioned above (Table S2). We aimed to prevent false positives by setting an interval greater than that of the apparent direct repeat (Table S5). For SINEs, we searched for TSDs at three intervals (Table S6).

### 2.3. Motif Discovery

Using a Perl script, we extracted 30 nucleotide-long sequences from respective copies of the SINE and LINE families, with 15 nucleotides upstream from the start of a 5′ direct repeat and 15 nucleotides downstream from the start of the repeat, from the genomic sequences obtained by the blastdbcmd (Section 2.2). The multiple expectation maximizations for motif elicitation (MEME) discovery algorithm [54] was applied to the TSD datasets. These motifs are represented as position-dependent character probability matrices that indicate the likelihood of each character appearing at each position in the pattern. We performed the MEME analysis (MEME suite 4.11.2 [55]) with the following parameters via the command-line version: sequence type, DNA; minimum motif width, 15; maximum motif width, 30; minimum sites per motif, N (number of analyzed TSDs) × 0.25; maximum sites per motif, N; type of model, zoops; and maximum number of motifs, 3. For SINEs from *Podarcis muralis* and *Pogona vitticeps*, we analyzed 2000 randomly selected TSDs. All available TSDs were used for the other species and LINEs. The most significant (lowest E-value) motif was selected for further analyses (Table S7). We identified a Bov-B motif using TSD datasets with maximum intervals in *Anolis carolinensis* (≥2999 bases) and *Bos taurus* (≥3699 bases) but not with smaller intervals (Table S5). For the other species, we used the TSD datasets with maximum intervals (Table S8).

### 2.4. Age Estimation of Bov-B

We estimated the mean evolutionary divergence over all Bov-B sequence pairs in a species using sequences with lengths > 3000 nucleotides. Multiple sequence alignment was performed with MAFFT version 7.511 [56], and then the number of base substitutions per site was calculated by averaging over all sequence pairs with molecular evolutionary genetics analysis version 11 (MEGA11) [57]. The codon positions included were 1st + 2nd + 3rd + Noncoding. All sites containing missing data or alignment gaps were removed from each sequence pair (pairwise deletions). We also estimated the Bov-B age for each species from the sequence divergence of all copies by multiplying half of the mean value from a representative copy (Section 2.2; Table S4) by a nucleotide substitution rate of 0.0013/site/million years [58,59,60].

### 2.5. Phylogenetic Analysis and Divergence Time Estimation

We conducted a phylogenetic analysis of the RTE-clade LINEs using the amino acid sequences of the ORF2 protein that includes the EN and RT domains. The alignment was performed using multiple sequence comparison by log-expectation implemented with MEGA11 and the following parameters: gap opening penalty, −2.90; gap extension penalty, 0.00; hydrophobicity multiplier, 1.20; clustering method, unweighted pair group method with arithmetic mean; and minimum diagonal length, 24. Next, we constructed phylogenetic trees in MEGA11 using the maximum likelihood method and a Jones–Taylor–Thornton matrix-based model, using the amino acid sequences of the ORF2 proteins from LINEs. These sequences were selected from those for which almost all the full-length data were available (sequence references are provided in Table S9). The bootstrap consensus tree was inferred from 500 replicates [61]. Overall, our analysis considered 1508 positions in the final dataset. Divergence time estimation was performed using the RelTime method implemented with MEGA11, using the monocot/eudicot divergence time of 140 MYA [62,63] and/or the Toxicofera (Anguimorpha and Iguania)/Serpentes divergence time of 184.6 MYA [64] as calibration constraints. A discrete gamma distribution was used to model the evolutionary rate differences between the sites. Phylogenetic analysis of squamate Bov-B LINEs was conducted using nucleotide sequences of almost full-length and partial (diverged in *I. braminus*) Bov-B LINEs. Phylogenetic trees were constructed in MEGA11 using the maximum likelihood method with 500 replicates.

## 3. Results

### 3.1. Long-Standing Activity of Reptilian Bov-B LINEs

To elucidate the genomic insertion signatures of the reptilian Bov-B LINE and associated SINE, we identified LINE and SINE family members in the genomes of squamates and Bov-B LINE family members in ruminants (Table 1 and Table S3). BLAST searches against the whole-genome sequences of 16 species (all nine squamates and six representative ruminants in Ensembl and *Indotyphlops braminus*) identified Bov-B copies for the respective species with significantly different numbers. Elapid snakes harbored relatively small numbers of Bov-B copies among squamates, while ruminants harbored a significantly greater number of Bov-B than squamates.

We also identified family members of the Sauria SINE via BLAST searches in many of the squamate species examined (Table 1 and Table S3). The copy number of Sauria SINE tended to be lower than that of Bov-B LINE in the same species. Among the surveyed species, *Podarcis muralis* and *Anolis carolinensis* harbored greater copy numbers of SINE than LINE. Such a successful SINE amplification in *A. carolinensis* was reported previously [65].

**Figure 2:**
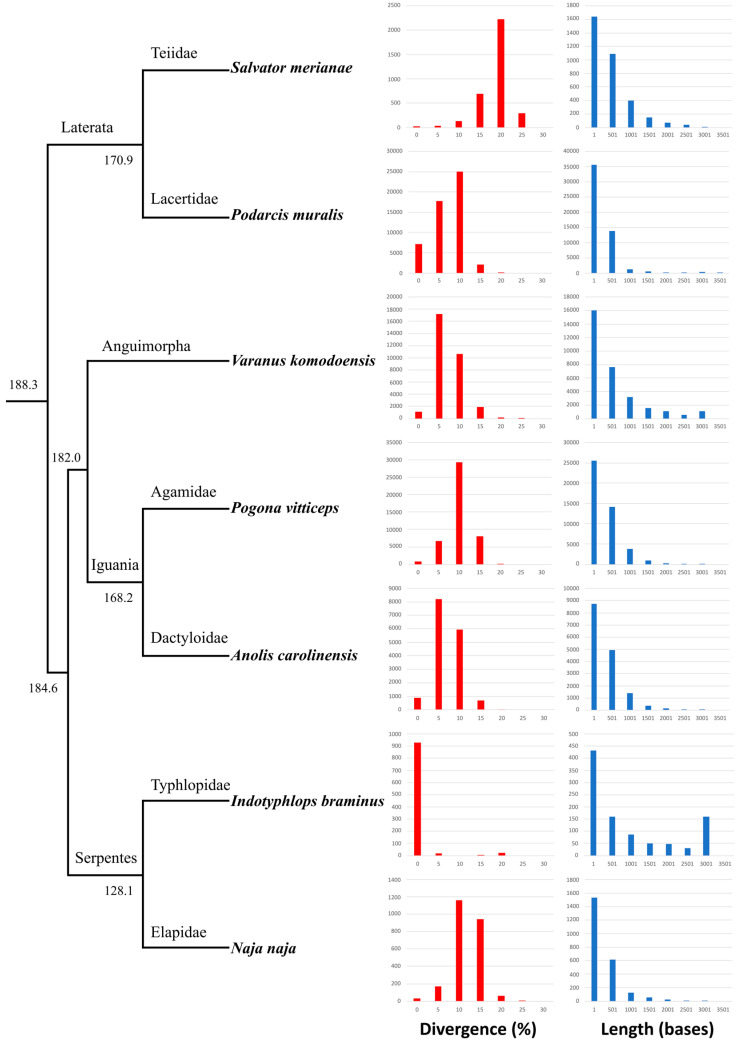
Varying sequence divergence of Bov-B LINEs across squamate species. The panels on the left indicate the frequency distribution of sequence divergence, and the panels on the right indicate the frequency distribution of sequence length. The vertical axis represents the number of LINE copies. All hits resulting from the second BLAST for each species were used to generate graphs. The phylogenetic relationships between higher-level squamate clades and their ages were obtained from Zheng and Wiens [64].

Figure 2 and Figure S2 present the frequency distributions of sequence divergence (extent of the difference between the query and copies) and sequence lengths for all LINE copies detected in each species. As commonly observed in other LINEs [27], we observed numerous truncated copies of the Bov-B LINE, which is approximately 3.5 kb in full length. Many of these copies were less than 1 kb in size (nucleotide sequence of Bov-B from *P. muralis*, with a length of >3 kb, is presented in Figure S3).

The sequence divergence of Bov-B LINEs varied across species. For instance, *S. merianae* exhibited a relatively large divergence, while *I. braminus* exhibited a very small divergence. Table 2 presents the sequence divergence and estimated ages of squamate Bov-B. Although absolute dating with a single substitution rate is generally difficult, we used a single substitution rate to estimate the ages since the nucleotide substitution rate among squamates presented relatively low variations [59]. The mean amplification time for each lineage ranged from 133 to 9 million years. Although most copies fell within the range of 110–40 MYA, some copies were over 150 MYA (*S. merianae*) or almost zero (*I. braminus*). These results suggest that the Bov-B LINE retained its retrotranspositional activity during squamate evolution. The Bov-B in *I. braminus* consisted of old copies with large divergence (mean of 21.4%) and young copies with small divergence (mean 1.4%) (Table 2 and Figure S4), with the estimated ages of the two groups at 134 million and 9 million years, respectively (Table 2).

**Figure 3:**
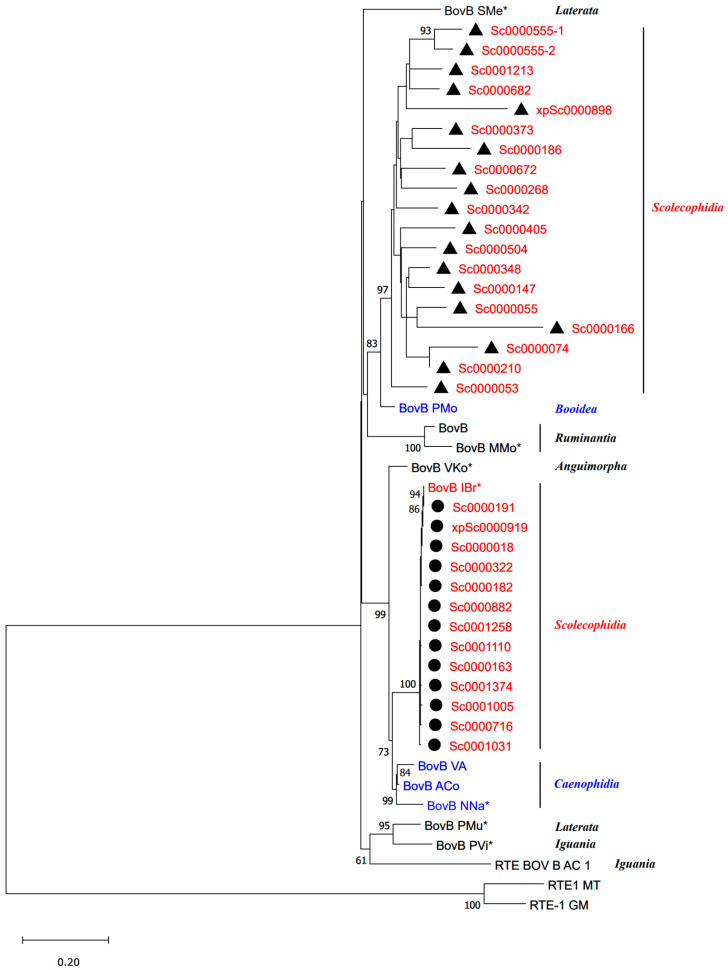
Phylogenetic relationships among squamate Bov-B LINEs. Bov-B LINEs from *Indotyphlops braminus* (*Scolecophidia*) with large and small divergence are represented by the filled triangle and circle, respectively. Snake Bov-B, other than that of *I. braminus* (in red), is highlighted in blue. Bov-B LINEs identified in this study are marked by asterisks (Table S4). Other LINEs were obtained from Repbase (Table S1). The phylogenetic tree was constructed using the maximum likelihood method with the nucleotide sequences of the Bov-B LINEs. Bootstrap analysis was performed with 500 replicates. Legume RTE-clade LINEs were used for an outgroup. SMe: *Salvator merianae*; PMo: *Python molurus*; VA: *Vipera ammodytes*; ACo: *Agkistrodon contortrix*; NNa: *Naja naja*; MT: *Medicago truncatula*; GM: *Glycine max*. Other abbreviations are presented in Table 4.

We analyzed phylogenetic relationships among squamate Bov-B copies, including those in the two *I. braminus* groups (Figure 3). The results indicated that squamate Bov-B LINEs possibly diverged into at least three distinct lineages and demonstrated that the old and young *I. braminus* copies belonged to two different lineages: one consisting of Bov-B copies from a lizard and Booidea and the other consisting of Bov-B copies from a lizard and Caenophidia snakes. Considering the squamate phylogeny (Figure 2), these results suggest that the origin of squamate Bov-B LINEs dates to before the divergence of Squamata lineages and that the Bov-B LINE was active during squamate evolution. Thus, the origin point could exceed 180 MYA.

### 3.2. Insertion Signature near the DNA Cleavage Site in Bov-B LINEs

Next, we searched for TSDs within the sequences from respective copies of the LINE and SINE families (Table 1, Tables S5 and S6). Young Bov-B copies from *I. braminus* exhibited conspicuous TSDs (Figure S5; a search result for Bov-B in *P. muralis* is presented in Figure S6). To investigate the DNA cleavage preference of Bov-B EN, we focused on 30 nucleotides around the first nucleotide (a DNA cleavage site) within the 5′ direct repeat (which constitutes the 5′-end of a TSD). A distinct insertion signature was observed around Bov-B TSDs in *I. braminus* and other species (Table 3). The signature was observed in species with a high proportion of copies exhibiting low divergence (less than 15%), often exceeding 81% of the copies (Table 3).

**Figure 4:**
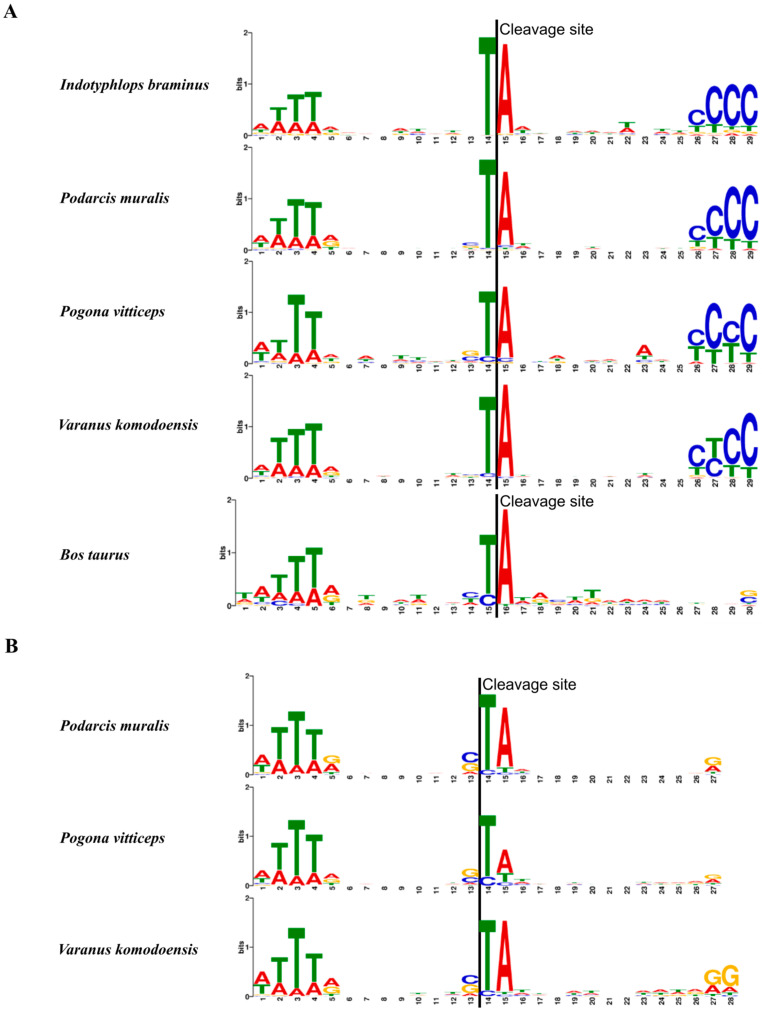
Insertion signatures of the Bov-B LINEs and Sauria SINEs. (**A**) Comparison of Bov-B LINE motifs across reptilian species and cows. The first DNA strand cleavage sites that correspond to a phosphodiester bond between the first nucleotide of a 5′ direct repeat and the adjacent 5′ flanking nucleotide on the complementary strand are indicated by a vertical line. The first nucleotides were predominantly adenine for all species analyzed. Three consecutive thymines are located at approximately ten nucleotides upstream. A stretch of cytosine at approximately 12 nucleotides downstream corresponds to the beginning of the reptilian Bov-B LINE full-length sequence (Figure S5). (**B**) Comparison of the SINE motifs across reptilian species. Sauria SINEs with a Bov-B-shared 3′-end from *P. muralis* (**upper**), *Pogona vitticeps* (**middle**) and *Varanus komodoensis* (**lower**). The first DNA strand cleavage sites are indicated by a vertical line. The first nucleotide in the TSDs was predominantly thymine for all species analyzed. The nucleotide at approximately 12 nucleotides downstream corresponds to the beginning of a Sauria SINE copy sequence. The vertical axis represents the information content of the site. Motifs with low scores for the Bov-B LINEs from *A. carolinensis* and *Moschus moschiferus* and for the Sauria SINEs from *I. braminus*, *Naja naja* and *Pseudonaja textilis* are presented in Figure S7. The MEME results for the Bov-B and Sauria SINE from *P. muralis* are presented in Figures S8 and S9, respectively. Detailed information on the discovered motif sites and E-values is provided in Table S7.

As presented in Figure 4A, Bov-B TSDs consistently started with adenine at approximately one helical pitch (ten nucleotides) downstream of the three thymine residues. Moreover, the 5′-end nucleotide next to adenine is typically thymine (Figure 4 and Figure S7). We detected TSDs in approximately 80% of the *I. braminus* copies (Table S5), with this motif observed in 108 out of 123 TSDs analyzed. This pattern with such high frequency likely did not occur by chance. We will henceforth refer to this insertion signature as “Tn-TA.” Tn-TA was observed in *I. braminus* and four lizard species (Table 3), and a clear Tn-TA pattern in Bov-B LINE was also observed in *Bos taurus* (Figure 4A).

### 3.3. Variation in the Tn-TA Pattern Between Bov-B LINEs and Sauria SINEs and Correlation with 3′-End Microsatellite-Like Sequences

We analyzed the nucleotide frequency around the first nucleotide of the 5′ direct repeats from Sauria SINEs identified in the genomes of three lizards and five snake species (Table 1 and Table S1). We observed that these SINEs exhibited the same Tn-TA pattern in lizards and the three snakes (Figure 4B and Figure S7; Tables S6 and S8). The first nucleotide of the TSDs from these SINEs was thymine, whereas that in Bov-B LINEs was adenine.

In our investigation, we observed distinct Tn-TA trends between the Bov-B LINEs and their associated Sauria SINEs in lizard species that included *P. muralis*, *P. vitticeps*, and *V. komodoensis* (Figure 4 and Figure S7; Table S8). A comparison of the Tn-TA patterns of LINEs and SINEs within the same species indicated that both contain three consecutive thymines, approximately 10 nucleotides upstream of the first nucleotide of the 5′ direct repeats. However, a crucial difference was noted: the first nucleotide was consistently adenine in the LINEs from all species but thymine in the SINEs (Figure 4 and Figure S7).

To investigate the cause of this difference, we examined the relationship between the first nucleotide of TSDs and microsatellite-like sequences at the 3′-ends of both LINEs and SINEs (Table 4 and Table S8). The reptilian Bov-B LINEs, where the first nucleotide is adenine, possessed a microsatellite-like sequence with consecutive adenine residues (CAA)_n_, except for *A. carolinensis*. In contrast, the Sauria SINEs, where the first nucleotide is thymine and the 3′-end sequence is shared with Bov-B LINEs, terminated in a sequence of consecutive thymine residues (ACCTTT)_n_.

### 3.4. Evolutionary Time Scale of the Tn-TA Containing RTE-Clade LINEs

**Figure 5:**
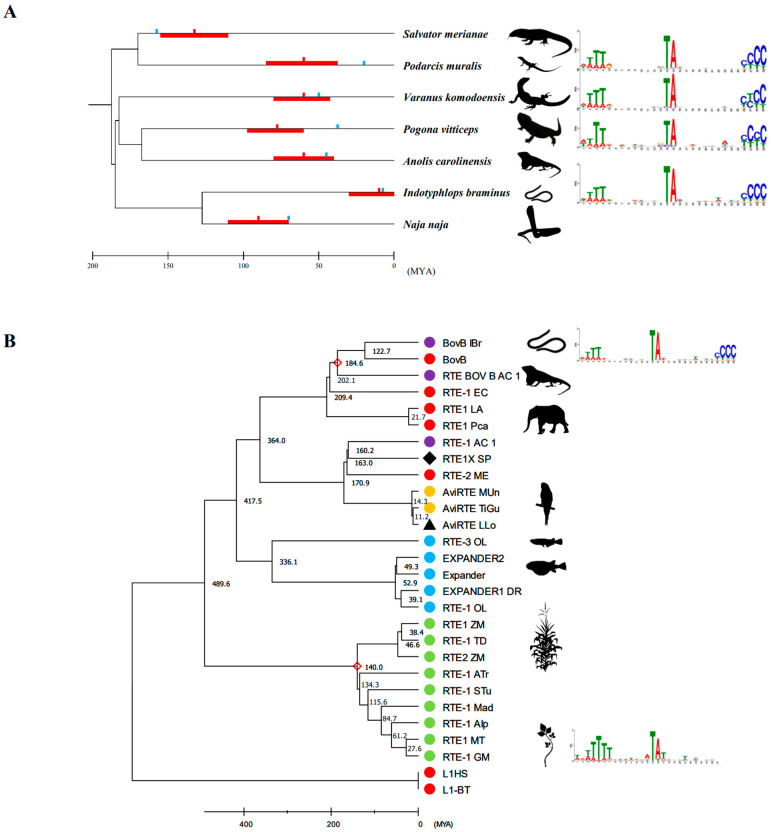
Evolutionary time scale of the Tn-TA-containing RTE-clade LINEs. (**A**) Amplification timing of squamate Bov-B LINEs that exhibit the Tn-TA pattern. Bov-B ages for respective lineages were estimated from the mean value of the sequence divergence of all copies and its standard deviation (red dot and thin rectangle) or average distance of overall sequence pairs of selected long copies (blue dot), using a nucleotide substitution rate of 0.0013/site/million years [58,59,60] (Table 2). Phylogenetic relationships between squamates and their divergence time were taken from [64]. (**B**) Phylogenetic relationships among the RTE-clade LINEs that exhibit the Tn-TA pattern. Species referenced in this study: Bov-B_IBr (*I. braminus*), Bov-B (*B. taurus*), and RTE_BOV_B_AC_1 (*A. carolinensis*). Species referenced in [29]: RTE-1_EC (horse), RTE1_LA (elephant), RTE1_MT (*Medicago*), and RTE-1_GM (soybean). Taxa in which the respective LINEs were identified are denoted as colored shapes: mammals (circles in red), reptiles (purple), birds (orange), fish (blue), plants (green), echinoderms (black square), and nematodes (black triangle). The phylogenetic tree was constructed using the maximum likelihood method with the amino acid sequences of the ORF2 protein of LINEs. The bootstrap consensus tree was inferred from 500 replicates (Figure S10). Mammalian L1 LINEs were used for an outgroup. Divergence time estimation was performed using the monocot/eudicot divergence time of 140 MYA [62,63] and Toxicofera/Serpentes divergence time of 184.6 MYA [64] as calibration constraints (red squares). The motif on the right of the plants was taken from the soybean SINE [29].

Figure 5A indicates the amplification timing of the squamate Bov-B LINEs estimated from the sequence divergence of each copy (Table 2). We identified a Tn-TA pattern in the respective Squamata lineages that diverged over 180 MYA. This suggests that the insertion signature or genomic integration machinery of LINEs is stably inherited along with these lineages, even if there are unusual HT events across squamate lineages. This is the first observation of stable and long-term inheritance of the integration machinery of RTE-clade LINEs.

Figure 5B presents a molecular phylogenetic tree that includes various RTE-clade LINEs, including the *I. braminus* Bov-B LINE that exhibits a Tn-TA pattern. It diverged into four major clades with statistical support, with the divergence almost parallel to the taxonomic groups, although their branching orders were not conclusive (Figure S10). The Tn-TA pattern has been observed in distantly related species, including plants and reptiles. Divergence time estimation with a calibration constraint of monocot/eudicot divergence suggested that the RTE lineages in angiosperms and vertebrates diverged over 411 MYA (411–1884 MYA; Figure 5B and Figure S11).

## 4. Discussion

### 4.1. Genomic Integration Machinery of Bov-B LINEs and Sauria SINEs

In the present study, we observed that the first nucleotide of the TSDs from the Bov-B LINE and Sauria SINE differed, with adenine in the Bov-B LINE and thymine in the Sauria SINE (Figure 4). Moreover, the Bov-B LINEs ended in (CAA)_n_ while the Sauria SINEs ended in (ACCTTT)_n_ (Table 4). A general consensus has not been established for the functional significance of the microsatellite-like sequences at the 3′ ends of LINEs and SINEs [66,67,68,69]. These repeat sequences could be required to ensure base pairing between the LINE RNA and exposed 3′ DNA for the initiation of reverse transcription [70]. However, Bov-B family members terminated with 3′ repeats are rarely flanked by target sequences that resemble the 3′ repeats (Figure S5). The different 3′ repeats between Bov-B and associated SINEs in the same species could be due to the different mechanisms for transcription termination of RNA Pol II and Pol III between LINEs and SINEs [71].

**Figure 6:**
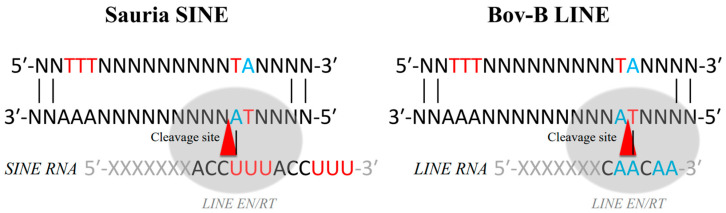
Model of the initial stage of genomic integration of Bov-B LINEs and Sauria SINEs. Consecutive residues within microsatellite-like repeats in the LINE RNA (AA: (**right**)) or SINE (UUU: (**left**)) influence whether Bov-B EN nicks one of the DNA strands at thymine or adenine. Moreover, they almost simultaneously (or alternatively) affect the efficiency of priming reverse transcription. DNA cleavage sites are indicated by a red arrowhead. EN: endonuclease, RT: reverse transcriptase.

The different DNA cleavage sites between LINEs and SINEs are explained by the hypothesis that consecutive residues (AA or UUU) within microsatellite-like repeats in LINE or SINE RNA influence whether Bov-B EN nicks one of the DNA strands at thymine or adenine and/or affect the efficiency of priming reverse transcription (Figure 6). These findings are consistent with previous results for RTE-clade LINEs and related SINEs [29].

TA dinucleotides may be primarily recognized by the three-dimensional structure of the EN domain in Bov-B ORF2 protein. Recent studies have shed light on the structural features of ORF2 proteins (including domains for both EN and RT) encoded in the *Bombyx mori* R2 LINE (R2Bm) and human L1 LINE [19,20,23,72]. The R2Bm-encoded protein recognizes a DNA sequence approximately 20 nucleotides upstream of the cleavage site, followed by first-strand cleavage [19,23]. A similar pattern was observed in the Bov-B LINE, where a thymine stretch was present upstream of the cleavage site. The Tn-TA pattern was observed in RTE-clade LINEs other than Bov-B, as well as in associated SINEs from several mammalian species (unpublished results). It is possible that most RTE-clade LINEs possess this signature. The thymine stretch is often three in animals, whereas it tends to be four or more in legumes [29]. Therefore, whether this difference in number indicates evolutionary changes in RTE ENs or simply reflects the different genomic landscapes of hosts, such as GC content, among taxa, should be determined. The integration machinery and role of microsatellite-like repeats can be evaluated using experimental approaches such as in vivo retrotransposition assays using RTE-clade LINEs as targets [69].

### 4.2. Ability of RTE-Clade LINEs to Propagate in a New Host Genome Following HT

Our results suggest that Tn-TA-containing RTE-clade LINEs in angiosperms and vertebrates diverged over 411 MYA (411–1884 MYA; Figure 5B and Figure S11). If this dates back before 600 MYA, the approximate origin of Metazoa, this deep branch raises the possibility that Tn-TA-containing RTE-clade LINEs originated from a common ancestor to both plants and animals. The machinery underlying the Tn-TA pattern, such as the characteristic EN, may be fundamental to the retrotransposition of RTE-clade LINEs and therefore might have been maintained over such a long evolutionary timescale. The insertion signature of RTE-clade LINEs from other species, such as fish and birds, remains to be elucidated. If the distribution of the Tn-TA-containing RTE-clade LINEs among taxa is patchy, it raises the possibility that this signature might be occasionally lost. Alternatively, complex evolutionary trajectories across species might account for their widespread distribution. As noted previously, an ancestor of plant RTE-clade LINEs likely underwent HT from ancient aphids or ancestral arthropods to angiosperms [16]. The long distance between angiosperm and vertebrate RTEs (Figure 5B) may reflect the divergence time between arthropods and vertebrates. Moreover, a probable HT event between reptiles (RTE-1_AC_1) and sea urchins (RTE1X_SP), as well as between birds and nematodes (AviRTE [4]), was observed in the phylogeny (Figure 5B). More extensive taxon sampling might have led to intermediates between reptiles and sea urchins. The missing link between plant and reptilian LINEs may include insects, fish, and avian LINEs and thus should be further investigated to provide insights into the trajectory of cross-species HT [4,5,16,73].

The Tn-TA pattern was observed in ruminants and snakes. The first nucleotide of the TSDs was also adenine in *B. taurus* (Figure 4A). However, the microsatellite-like sequences of the Bov-B LINEs differed between *B. taurus* and *I. braminus*, with (CTGAA)_n_ and (CAA)_n_ repeats, respectively (Table 4). This difference suggests vestiges of adaptation to a new genome following HT. Specifically, an ancient snake Bov-B LINE may have acquired a new microsatellite at the 3′-end in the genome of an ancestor of ruminants after HT, thus allowing the LINE to propagate in the host genome.

Although several reports have been published on possible HT vectors in RTE-clade LINEs [74], the mechanism underlying the increased ability of RTE-clade LINEs to propagate in a new host genome following HT relative to that observed for L1-clade LINEs remains unclear. Environmental stressors, such as chemical agents, physical agents (radiation), and experiential factors (e.g., environmental light/dark cycles), can affect the retrotransposition of the human L1 LINE [75], whereas such external factors for RTE-clade LINEs have not yet been reported. RTE-clade LINEs generally contain a single ORF and lack what corresponds to the first ORF of the L1-clade LINEs. Because the ORF1 protein from human L1 interacts with host proteins, which in turn affect L1’s activity [76,77,78], the lack of the ORF1 protein may release RTEs from the regulation by host factors and facilitate their HT. Furthermore, for LINEs with indiscriminate integration sites, separation of the DNA cleavage site and ordinary recognition motif within a short distance, Tn-TA may have some advantages, such as providing LINE EN with flexibility to nick DNA. Thus, whether this characteristic is also present in LINEs other than in RTEs should be further investigated. Future TSD surveys for distinct clades of LINEs and SINEs may lead to the discovery of another insertion signature, which could enhance our understanding of the integration strategies of LINEs and SINEs and their evolutionary impact on host genomes.

## 5. Conclusions

We proposed a model for the genomic integration machinery of Bov-B LINEs and Sauria SINEs, which can be evaluated experimentally. We also observed long-term activity of Bov-B LINE with a characteristic insertion signature. Although a complex evolutionary trajectory across species is plausible, the long evolutionary distances of Tn-TA-containing LINEs among species suggest an ancient origin for the mechanism underlying this signature. Despite their large occupancy in eukaryotic genomes, the mechanism of LINEs with indiscriminate integration sites, except for L1s and RTEs, remains poorly understood. The accurate detection of TSDs and the discovery of novel insertion signatures for distinct clades of LINEs and SINEs could contribute to a better understanding of the evolutionary and functional impacts of these elements.

## Figures and Tables

**Table 1:** Copy Numbers of the LINEs and SINEs and Number of Analyzed TSDs.

Retroposons	Species	Common Name	Genome Size (Mb) ^(1)^	Number of Hits ^(2)^	TSD Interval (Bases) ^(3)^	TSDs ^(4)^
Bov-B LINE	*Salvator merianae*	Argentine black and white tegu	2026	3393	≥2999	1
	*Podarcis muralis*	Common wall lizard	1511	52,041	≥2999	353
	*Pogona vitticeps*	Central bearded dragon	1816	45,093	≥3099	62
	*Anolis carolinensis*	Green anole	1799	15,740	≥2999	41
	*Varanus komodoensis*	Komodo dragon	1508	31,077	≥2999	509
	*Indotyphlops braminus*	Brahminy blindsnake	1856	968	≥2999	123
	*Naja naja*	Indian cobra	1769	2370	≥2999	3
	*Pseudonaja textilis*	Eastern brown snake	1590	1919	≥2999	2
	*Notechis scutatus*	Mainland tiger snake	1666	1981	≥2999	2
	*Laticauda laticaudata*	Blue-lipped sea krait	1559	668	≥2999	3
	*Bos taurus*	Cow	2716	365,688	≥3699	153
	*Bison bison bison*	American bison	2954	346,426	≥3499	82
	*Capra hircus*	Goat	2923	292,528	≥3499	487
	*Ovis aries*	Sheep	2870	334,914	≥3499	408
	*Moschus moschiferus*	Siberian musk deer	3070	357,380	≥3499	105
	*Cervus hanglu yarkandensis*	Yarkand deer	2594	219,829	≥3499	38
Sauria SINE	*Salvator merianae*	Argentine black and white tegu	2026	0	n/a	n/a
	*Podarcis muralis*	Common wall lizard	1511	77,228	≥99	40,838
	*Pogona vitticeps*	Central bearded dragon	1816	10,602	≥99	5807
	*Anolis carolinensis*	Green anole	1799	^(5)^ 78,442	≥99	^(5)^ 33,597
	*Varanus komodoensis*	Komodo dragon	1508	845	≥99	378
	*Indotyphlops braminus*	Brahminy blindsnake	1856	497	≥99	129
	*Naja naja*	Indian cobra	1769	1041	≥99	233
	*Pseudonaja textilis*	Eastern brown snake	1590	986	≥99	227
	*Notechis scutatus*	Mainland tiger snake	1666	408	≥99	86
	*Laticauda laticaudata*	Blue-lipped sea krait	1559	448	≥99	90

^(1)^ Number of nucleotides in the genome assembly. ^(2)^ Number of LINE and SINE copies was determined based on a second BLAST search. The initial survey results with a specific sequence as the query are presented in Table S3. ^(3)^ Interval of the 5′ and 3′ direct repeats used for each TSD search. ^(4)^ Number of TSDs with the interval indicated on the left. The number of direct repeats detected at different intervals for each species is presented in Table S5 (for LINE) and Table S6 (SINE). ^(5)^ Nishiyama E, Ohshima K. [29]. n/a: Not available.

**Table 2:** Sequence Divergence and Estimated Ages of Squamate Bov-B.

Species	Mean Pairwise Divergence (%) ^(1)^	Mean Divergence from a Copy (% ± SD) ^(2)^	Age (MYA) ^(3)^
*Salvator merianae*	25.0	21.3 ± 3.6	133 (111–156)
*Podarcis muralis*	3.0	9.7 ± 3.7	61 (37–84)
*Pogona vitticeps*	6.0	12.6 ± 2.9	78 (60–97)
*Anolis carolinensis*	7.0	9.5 ± 3.1	60 (40–79)
*Varanus komodoensis*	8.0	9.8 ± 3.2	61 (42–81)
*Indotyphlops braminus* ^(4)^	1.0	1.4 ± 3.3	9 (0–30)
*Indotyphlops braminus* ^(5)^	n/a	21.4 ± 1.6	134 (124–144)
*Naja naja*	11.0	14.3 ± 3.3	89 (69–110)
*Pseudonaja textilis*	11.0	14.8 ± 3.0	92 (74–111)
*Notechis scutatus*	11.0	13.2 ± 2.9	82 (64–101)
*Laticauda laticaudata*	15.0	15.2 ± 2.4	95 (80–110)

^(1)^ Mean pairwise divergence between copies of >3000 nucleotides. ^(2)^ Mean divergence of squamate Bov-B copies from a representative Bov-B copy (Table S4). The representative squamate copies appear in Figure 3. SD: standard deviation. ^(3)^ Bov-B age for a species was estimated from the mean sequence divergence of all copies using a nucleotide substitution rate of 0.0013 per site/million years [58,59,60]. ^(4)^ A mean of 159 copies with length > 3 kb (1) and all 968 copies (2). ^(5)^ Mean of the 23 copies with the largest divergence (Subfamily II; Figure S4).

**Table 3:** Insertion Signature of Bov-B LINEs and Overall Proportion of Copies Exhibiting Low Divergence.

Species	Length > 3 kb ^(1)^	Divergence < 0.15 ^(2)^	Tn-TA Trend ^(3)^
*Salvator merianae*	0.003 (11)	0.054 (183)	−
*Podarcis muralis*	0.008 (435)	0.960 (49,938)	+(265/353)
*Pogona vitticeps*	0.004 (164)	0.815 (36,760)	+(37/62)
*Anolis carolinensis*	0.004 (65)	0.954 (15,020)	+(37/41) ^(4)^
*Varanus komodoensis*	0.035 (1076)	0.934 (29,019)	+(313/509)
*Indotyphlops braminus*	0.164 (159)	0.976 (945)	+(108/123)
*Naja naja*	0.004 (9)	0.574 (1360)	−
*Pseudonaja textilis*	0.003 (5)	0.492 (944)	−
*Notechis scutatus*	0.004 (8)	0.767 (1519)	−
*Laticauda laticaudata*	0.007 (5)	0.439 (293)	−
*Bos taurus*	0.022 (8199)	0.850 (310,773)	+(42/153)
*Bison bison bison*	0.008 (2632)	0.837 (289,932)	−
*Capra hircus*	0.032 (9244)	0.702 (205,434)	−
*Ovis aries*	0.029 (9554)	0.593 (198,506)	−
*Moschus moschiferus*	0.012 (4265)	0.806 (287,877)	+/−(27/105) ^(5)^
*Cervus hanglu yarkandensis*	0.023 (5005)	0.599 (131,607)	−

^(1)^ Ratio of the number of copies with length > 3000 nucleotides to the total number of copies. The number of copies with length > 3 kb is indicated in parentheses. ^(2)^ Ratio of the number of copies with sequence divergence less than 0.15 to the total number of copies. The number of copies with sequence divergence < 0.15 is indicated in parentheses. ^(3)^ Species exhibiting a Tn-TA pattern in the TSDs are indicated by +. The number of sites contributing to the construction of the motif and number of analyzed TSD sites are indicated in parentheses. ^(4)^ Motif was statistically significant; however, the E-value was not significantly low (Figure S7A, Tables S5 and S7). ^(5)^ A Tn-TA-like pattern was observed; however, the E-value was not significant (Figure S7A, Table S7).

**Table 4:** Correlation of 3′-end Microsatellite-Like Sequences and First Nucleotide of TSDs.

	Name	References	Species	3′Repeat	TSD ^(1)^
LINE	Bov-B_PMu	This study	*Podarcis muralis*	(CAA)_2–6_	A
	Bov-B_PVi	This study	*Pogona vitticeps*	(CAA)_3_/(CA)_4–8_	A
	RTE_BOV_B_AC_1	Repbase	*Anolis carolinensis*	(GCA)_2–4_	A [29]
	Bov-B_VKo	This study	*Varanus komodoensis*	(CAA)_2–4_	A
	Bov-B_IBr	This study	*Indotyphlops braminus*	(CAA)_2–5_	A
	Bov-B (BovB)	Repbase	*Bos taurus*	(CTGAA)_3–5_/(CTGAT)_3–6_	A [29]
	Bov-B_MMo	This study	*Moschus moschiferus*	(CTGAA)_3–4_	A
SINE	Sauria_POM	Repbase; Piskurek et al. [49]	*Podarcis muralis*	(ACCTTT)_1–2_	T
	Sauria_PVi	This study	*Pogona vitticeps*	(ACCTTT)_1–3_	T
	Sauria_ACA	Repbase; Piskurek et al. [49]	*Anolis carolinensis*	(ACCTTT)_2–4_	T [29]
	Sauria_VKo	This study	*Varanus komodoens*	(ACCTTT)_1–3_	T
	Sauria_IBr	This study	*Indotyphlops braminus*	(ACCTTT)_1–2_	T
	Sauria_NNa	This study	*Naja naja*	(ACCTTT)_1–2_	T
	Sauria_PTe	This study	*Pseudonaja textilis*	(ACCTTT)_1–3_	T

^(1)^ Microsatellite-like sequences at 3′-ends of the SINEs and LINEs consist of a stretch of T or A along with other nucleotides. The first nucleotides of TSDs and repeat nucleotides within the microsatellite-like sequences are consistent across many species.

## Data Availability

Data are contained within this article and Supplementary Materials.

## Supplementary Materials

The following supporting information can be downloaded at: https://www.mdpi.com/article/10.3390/biology15120927/s1, Table S1: Genome sequences and LINE/SINE sequences used in this study; Table S2: Query sequences used for the initial BLAST search and their internal direct repeats; Table S3: Genome survey for the Bov-B and Sauria SINE with the initial query sequences; Table S4: Query sequences used for the second BLAST search; Table S5: Copy numbers of the Bov-B and the number of direct repeats detected at different intervals; Table S6: The number of direct repeats detected beside Sauria SINEs at different intervals; Table S7: Discovered motif sites and statistical significance of motifs; Table S8: Comparison of Tn-TA trends and the first nucleotides of TSDs between LINEs and SINEs; Table S9: LINE sequences used in phylogenetic analysis. Figure S1: Python script for TSD search; Figure S2: Frequency distributions of sequence divergence and sequence length of LINE copies across snake and ruminant species; Figure S3: Sequence comparisons among Bov-B from *Podarcis muralis* with a length > 3 kb; Figure S4: Frequency distributions of sequence divergence and sequence length in the two *Indotyphlops braminus* Bov-B groups; Figure S5: Sequence comparisons among young *I. braminus* Bov-B copies and their TSDs; Figure S6: TSD search results with an interval of ≥2999 bases for Bov-B from *P. muralis*; Figure S7: Comparisons of the LINE/SINE motifs among different TSD intervals; Figure S8: MEME results for Bov-B from *P. muralis*; Figure S9: MEME results for Sauria SINE from *P. muralis*; Figure S10: ML tree of RTE-clade LINEs and bootstrap consensus tree; Figure S11: Estimation of the divergence time with different calibration constraints.
