# Supplementary material for: Long-Standing Activity with Characteristic Genomic Insertion Signatures in Reptilian Bov-B LINEs and Associated Sauria SINEs
Source: Biology (Basel). 2026 Jun 13;15(12):927. doi: 10.3390/biology15120927 (PMC13295347; doi:10.3390/biology15120927)
Supplement: Supplementary file 1 [file biology-15-00927-s001.zip › Supplemental_Fig 2-4-5-7-10-11.pdf]

Fig. S2

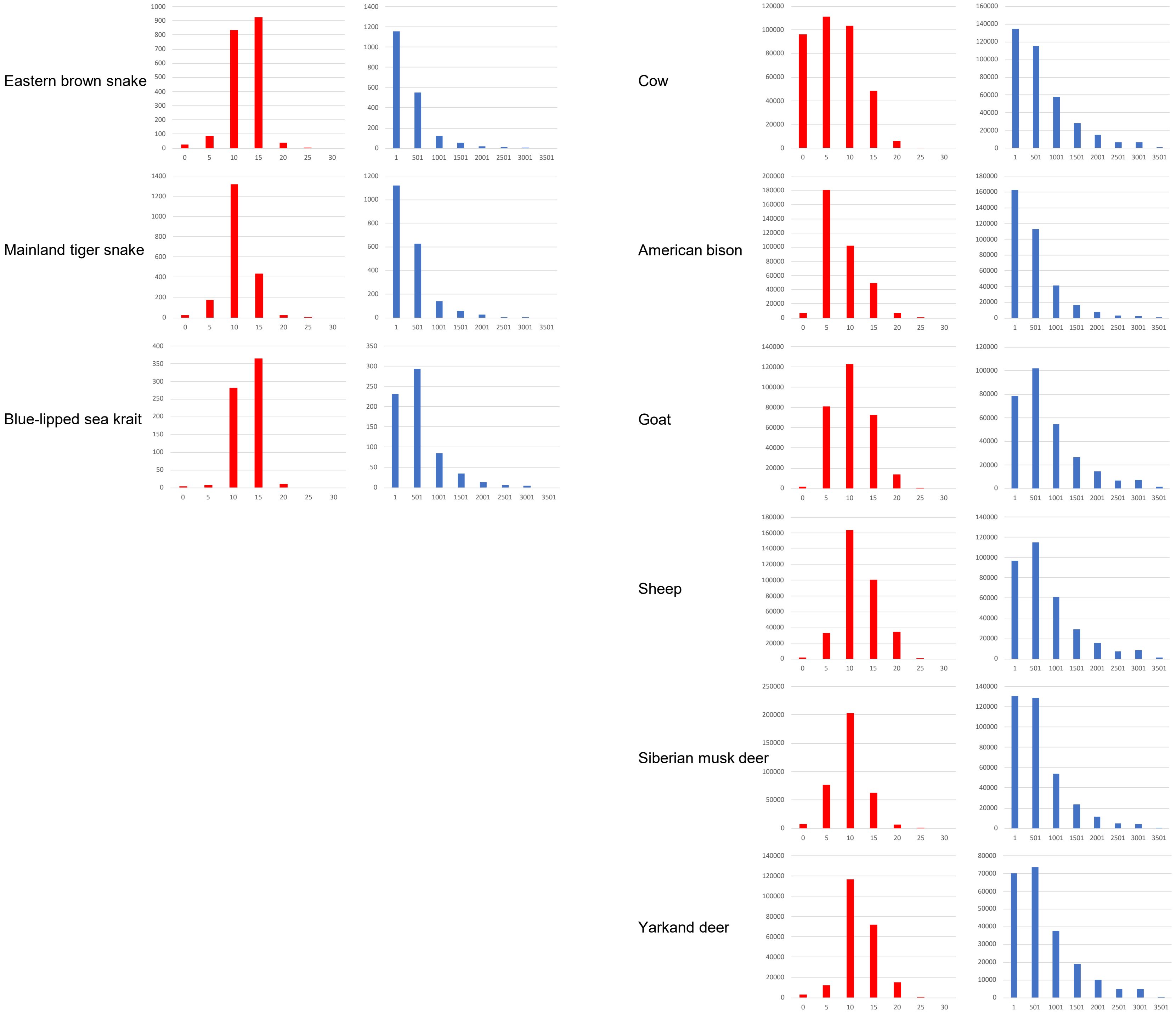
Eastern brown snake
Mainland tiger snake
Blue-lipped sea krait
Cow
American bison
Goat
Sheep
Siberian musk deer
Yarkand deer

Fig. S4

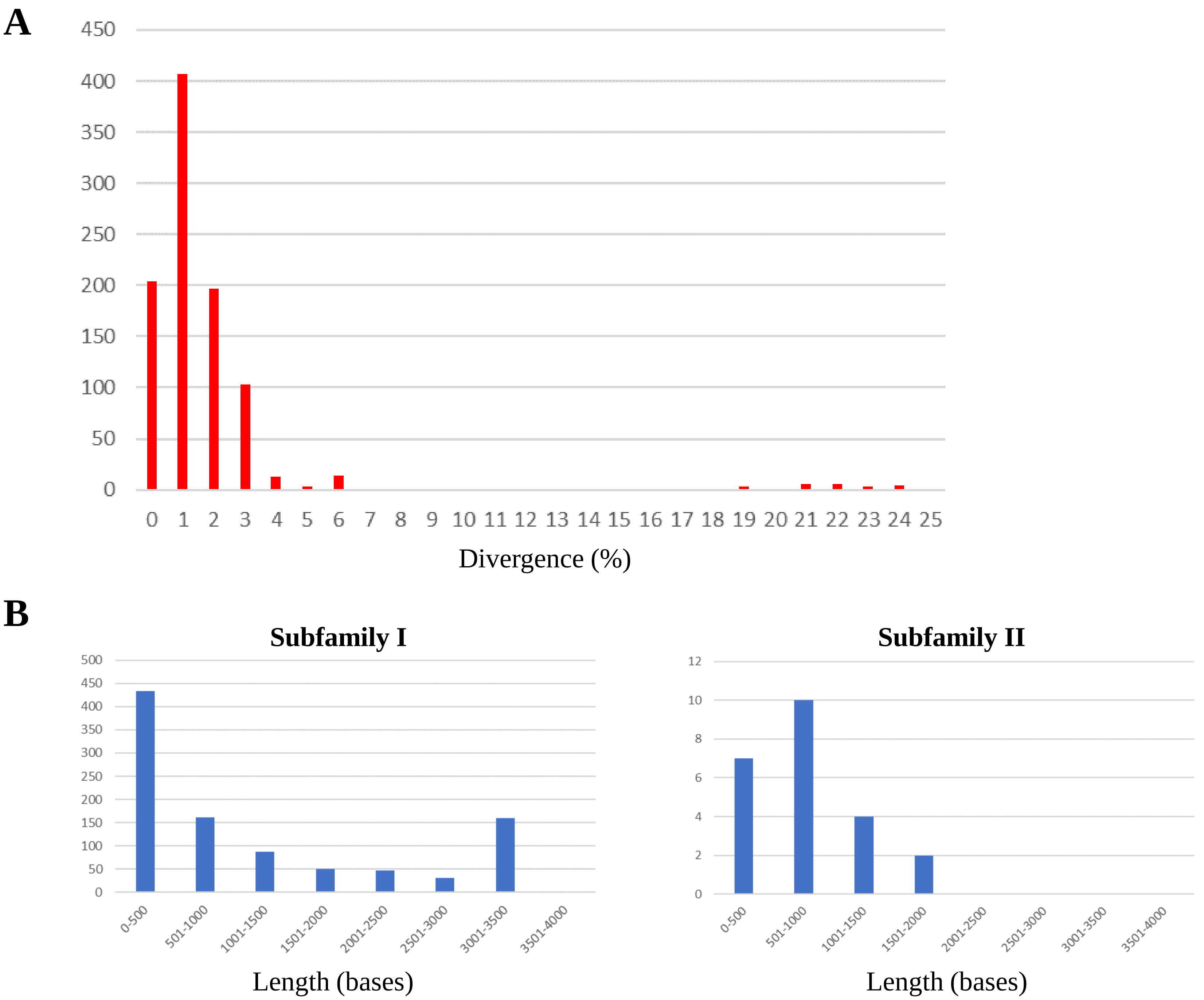
A
450
400
350
300
250
200
150
100
50
0
0 1 2 3 4 5 6 7 8 9 10 11 12 13 14 15 16 17 18 19 20 21 22 23 24 25
Divergence (%)
B
Subfamily I
500
450
400
350
300
250
200
150
100
50
0
0-500
501-1000
1001-1500
1501-2000
2001-2500
2501-3000
3001-3500
3501-4000
Length (bases)
Subfamily II
12
10
8
6
4
2
0
0-500
501-1000
1001-1500
1501-2000
2001-2500
2501-3000
3001-3500
3501-4000
Length (bases)

Fig. S5

5′ flanking | 5′ direct repeat | *Indotyphlops braminus* Bov-B (c.a. 3220 bases) | 3′ direct repeat | 3′ flanking

```
Sc0000182   ctacaaatgtccactagtctaatgtccatttcCCCCTTCATGGATCGCTGCCTTGCCGGGGCGAAGGGGCTT GATGGGTCGGACACGACTTCGCAACTAACAACAACAACAACaatgtccatttcttatgtttcttgggccaagcaactctc
xpSc0000919 tccaattctgttaaaatagatttcctctaagtcgtcttCATGGATCGCTGCCTTGCCGGGGCGAAGGGGCTT GATGGGTCGGACACGACTTCGCAACTAACAACAACAACAACaagtcgtcttttgctctcttttatttatctactgatcaat
Sc0000018   atcctgatttgaaaactgctaagatgaatagtCCCCTTCATGGATCGCTGCCTTGCCGGGGCGAAGGGGCTT GATGGGTCGGACACGACTTCGCAACTAACAACAACAACAaagatgaatagttcctgtgtgtttggaagcatcggtgatgg
Sc0001031   ctgtttttaaacttagtctatatacgatatatCCCCTTCATGGATCGCTGCCTTGCCGGGGCGAAGGGGCTT GATGGGTCGGACACGACTTCGCAACTAACAACAACAACAAatatacgatatataccatttccgacatagtggttcctgaa
Sc0001110   ttcaaaactttgtatctttatacttgtggactCCCCTTCATGGATCGCTGCCTTGCCGGGGCGAAGGGGCTT GATGGGTCGGACACGACTTCGCAACTAACAACAACAacttgtggactaatactgtcatggtaattactattcaatacatc
Sc0000322   acttctgaattggacagaactagctctctggtcCCCTTCATGGATCGCTGCCTTGCCGGGGCGGAGGGGCTT GATGGGTCGGACACGACTTCGCAACTAACAACAACAACagctctctggtcaccaccaagaactatttaccggattttgtt
Sc0001258   gtctacattattagctccaatatatatcagcgCCCCTTCATGGATCGCTGCCTTGCCGGGGCGAAGGGGCTT GATGGGTCGGACACGACTTCGCAACTAACAACAACAACAACAACatatatcagcgactccatcttgacaatcgcacagta
Sc0000716   gtaaaccaatatggtataatgtacatgtccatCCCCTTCATGGATCGCTGCCTTGCCGGGGCGAAGGGGCTT GATGGGTCGGACACGACTTCGCAACTAACAACAACAACACAttttatatttatagtcaagtcactatccccaccacgaca
Sc0001374   tactgaaaataaagaCCCTTCATGGATCGCTGCCCCTTCATGGATCGCTGCCTTGCCGGGGCGAAGGGGCTT GATGGGTCGGACACGACTTCGCAACTAACAACAACAACAACaaagagctgaagctagagaaagcacaagtcccagaaaag
Sc0001005   gatgtatgtttgtatgcttatatattaaAAAACCCCTTCATGGATCGCTGCCTTGCCGGGGCGAAGGGGCTT GATGGGTCGGACACGACTTCGCAACTAACAACAACAAAatattaataaaacatttttaaaaaatgtttgttcattattc
Sc0000163   atctcagataagggcacctgtatcttcttcttCCCCTTCATGGATCGCTGCCTTGCCGGGGCGAAGGGGCTT GATGGGTCGGACACGACTTCGCAACTAACAACAAAatcttcttcttgactgctctgtaccacatatccaatccacagcta
Sc0000191   cattataatgttggcatctctattttctattccCCCTTCATGGATCGCTGCCTTGCCGGGGCGAAGGGGCTT GATGGGTCGGACACGACTTCGCAACTAACAACAACAAAattttctattccatttttataattcctaacattctatttgc
Sc0000882   gtagataattaacccaatcataagaacataagCCCCTTCATGGATCGCTGCCTTGCCGGGGCGAAGGGGCTT GATGGGTCGGACACGACTTCGCAACTAACAACAACAACaagaacataagagatgccttgctggatcggattcttggtcca
```

Fig. S7

A

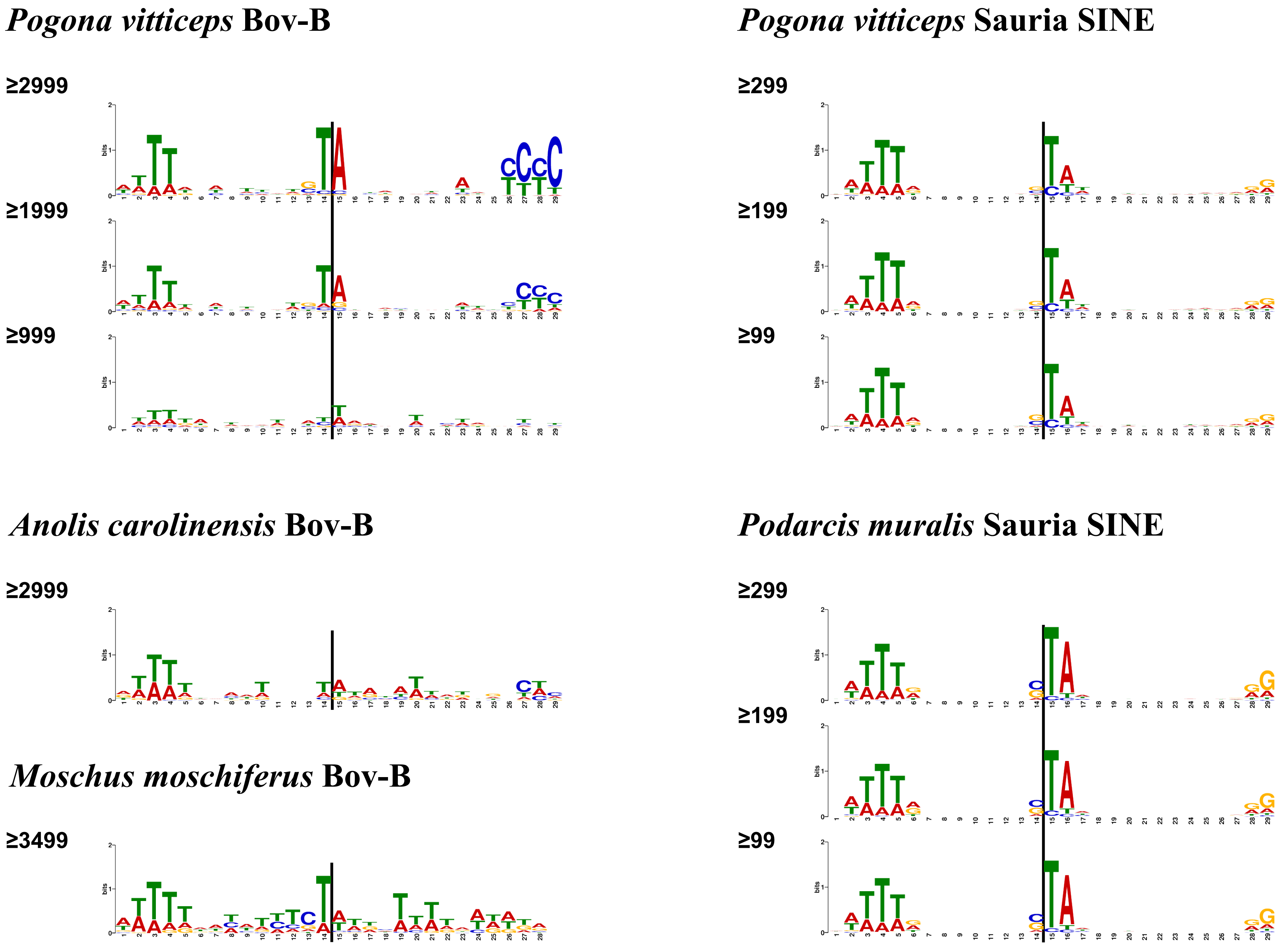
Pogona vitticeps Bov-B
≥2999
≥1999
≥999
Pogona vitticeps Sauria SINE
≥299
≥199
≥99
Anolis carolinensis Bov-B
≥2999
Moschus moschiferus Bov-B
≥3499
Podarcis muralis Sauria SINE
≥299
≥199
≥99

Fig. S7

B

Varanus komodoensis SINE

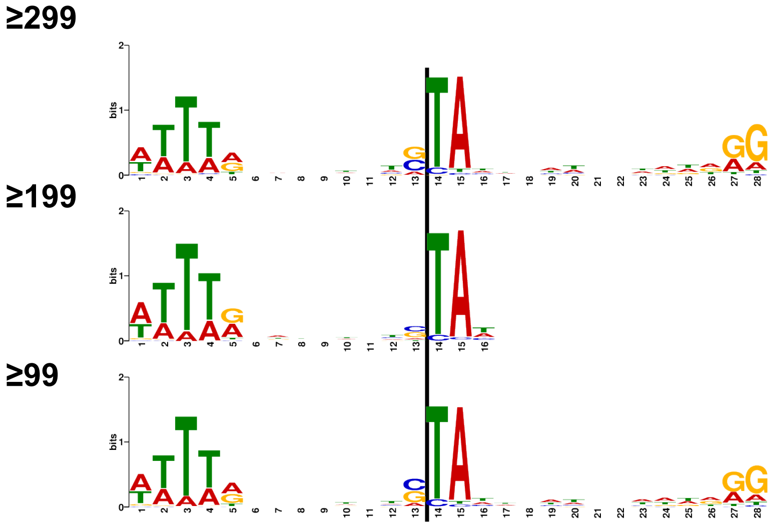

Indotyphlops braminus SINE

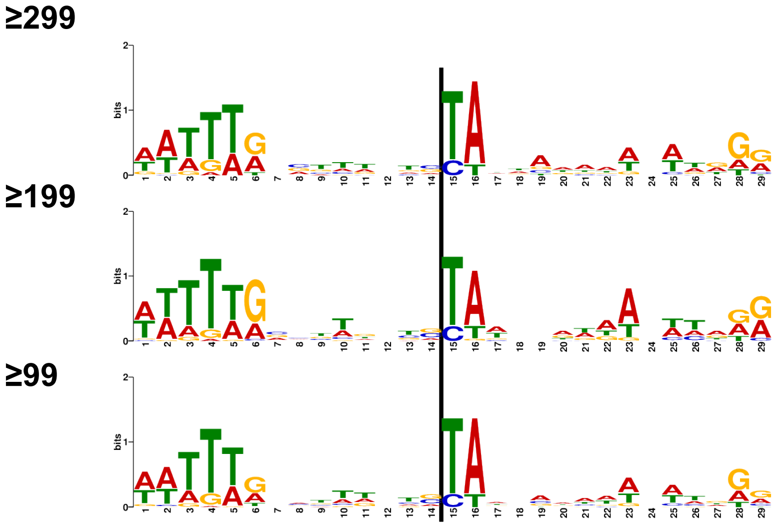

Naja naja SINE

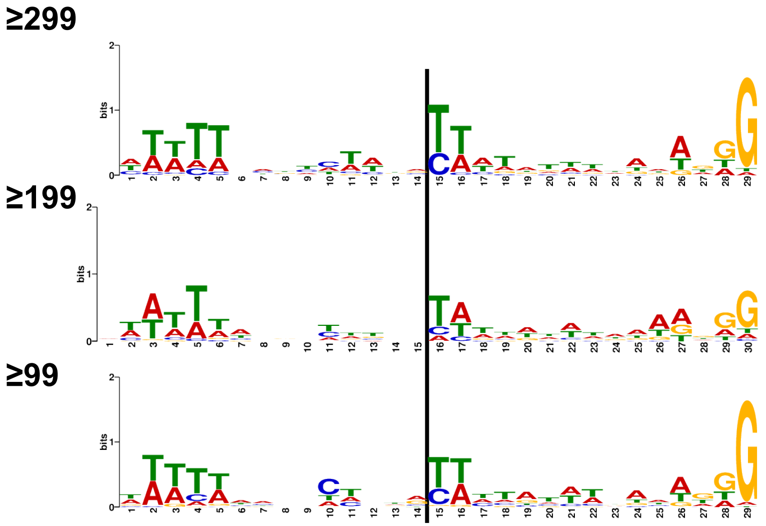

Pseudonaja textilis SINE

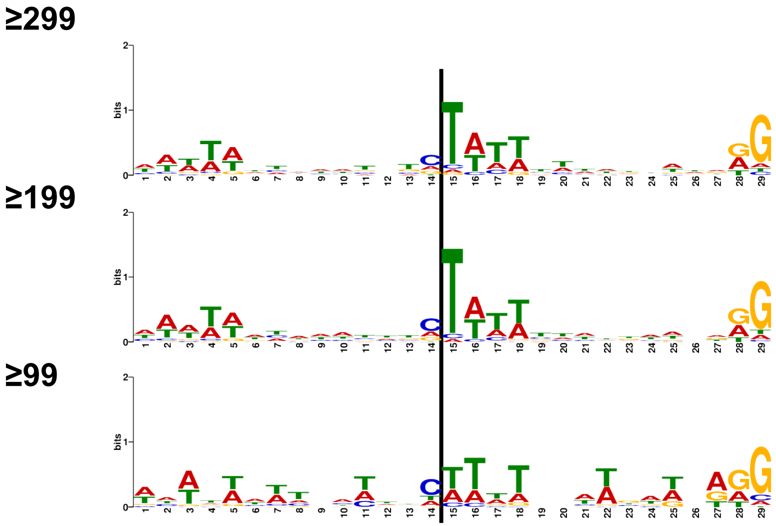

Fig. S10

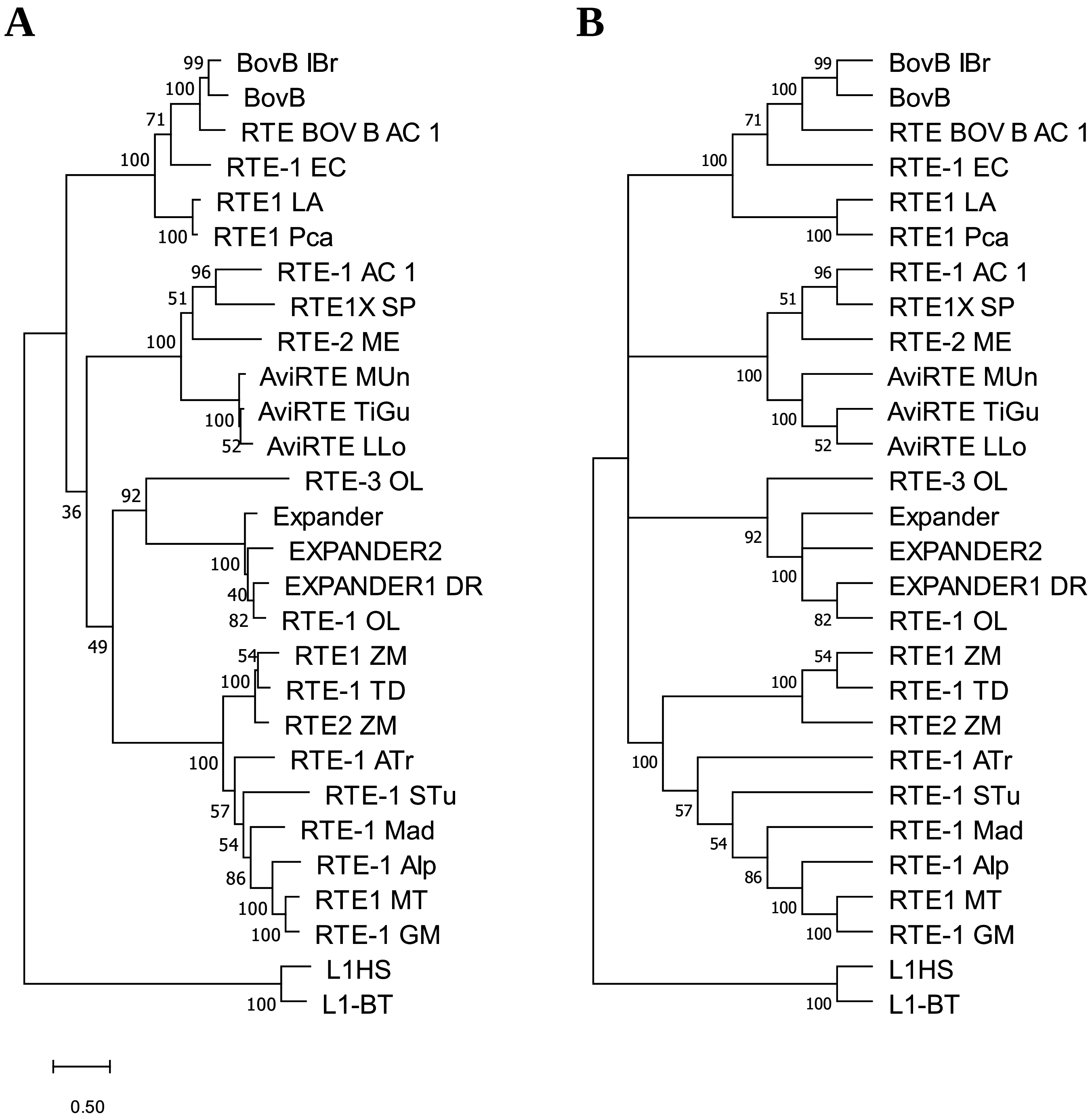
A
B
BovB IBr
BovB
RTE BOV B AC 1
RTE-1 EC
RTE1 LA
RTE1 Pca
RTE-1 AC 1
RTE1X SP
RTE-2 ME
AviRTE MUn
AviRTE TiGu
AviRTE LLo
RTE-3 OL
Expander
EXPANDER2
EXPANDER1 DR
RTE-1 OL
RTE1 ZM
RTE-1 TD
RTE2 ZM
RTE-1 ATr
RTE-1 STu
RTE-1 Mad
RTE-1 Alp
RTE1 MT
RTE-1 GM
L1HS
L1-BT
0.50

Fig. S11

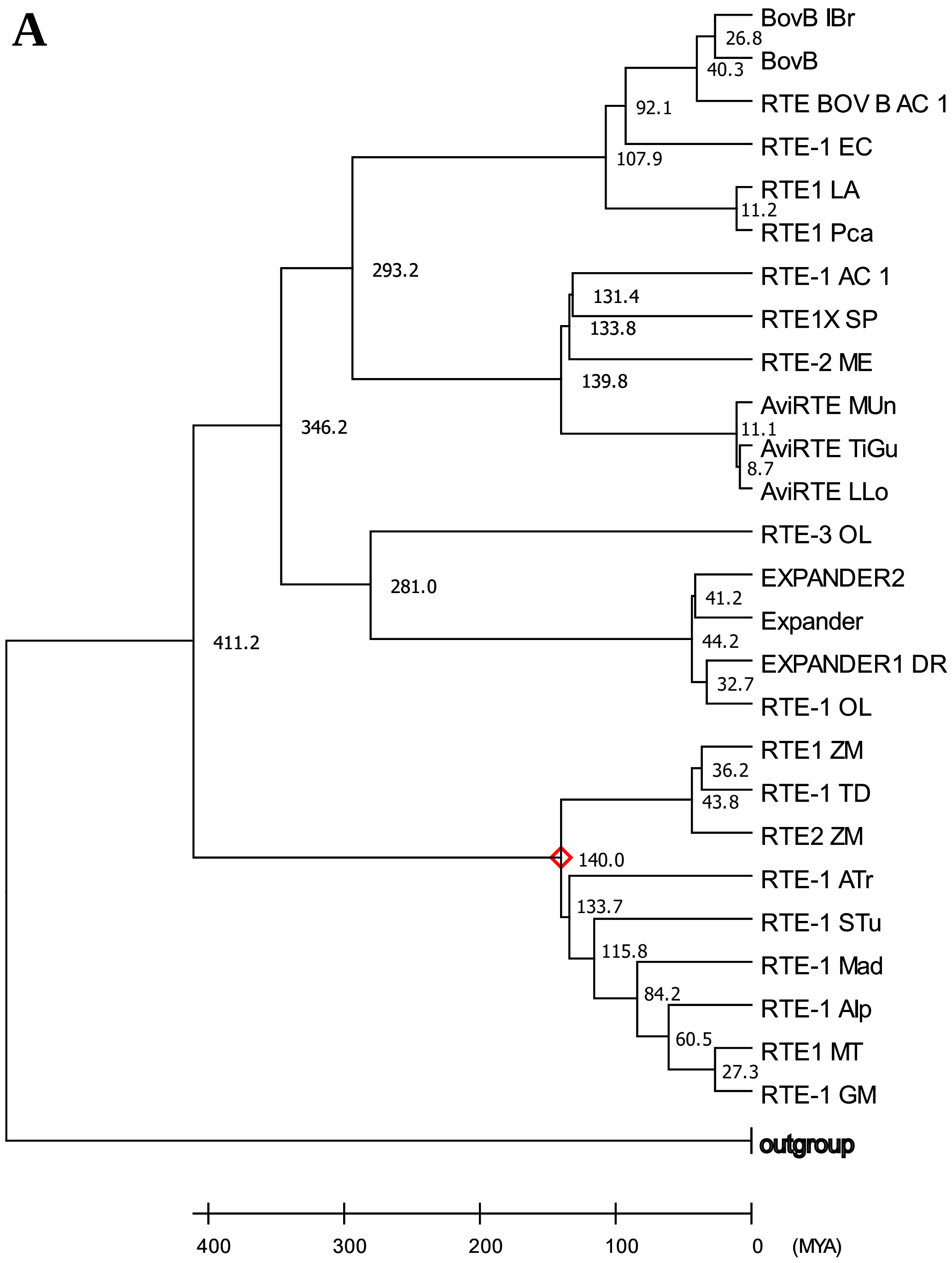
A
BovB IBr
26.8
BovB
40.3
RTE BOV B AC 1
92.1
RTE-1 EC
107.9
RTE1 LA
11.2
RTE1 Pca
293.2
RTE-1 AC 1
131.4
RTE1X SP
133.8
RTE-2 ME
139.8
AviRTE MUn
11.1
AviRTE TiGu
8.7
AviRTE LLo
346.2
RTE-3 OL
281.0
EXPANDER2
41.2
Expander
44.2
EXPANDER1 DR
32.7
RTE-1 OL
411.2
RTE1 ZM
36.2
RTE-1 TD
43.8
RTE2 ZM
140.0
RTE-1 ATr
133.7
RTE-1 STu
115.8
RTE-1 Mad
84.2
RTE-1 Alp
60.5
RTE1 MT
27.3
RTE-1 GM
outgroup
400
300
200
100
0
(MYA)

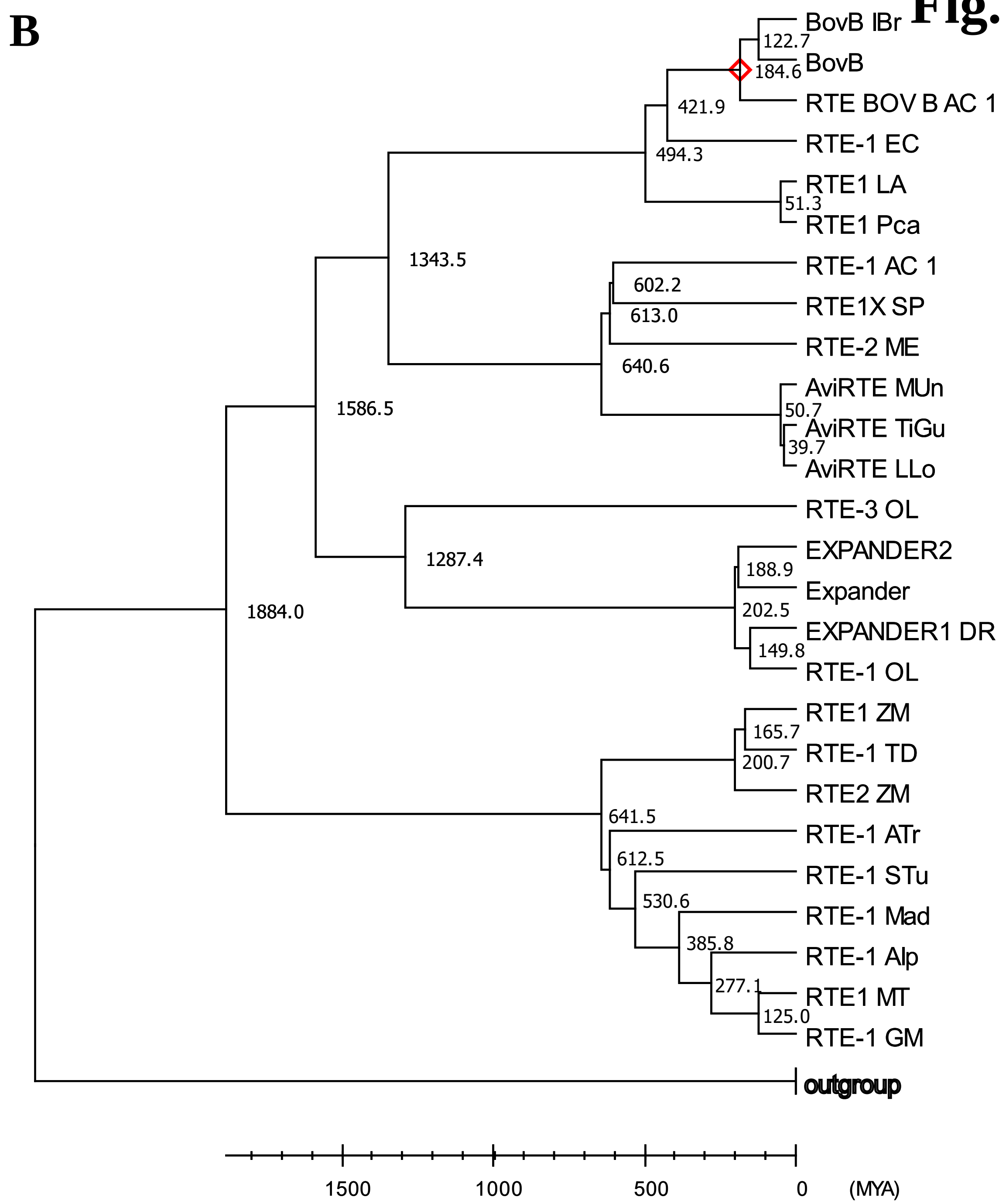
B
BovB IBr
122.7
BovB
184.6
RTE BOV B AC 1
421.9
RTE-1 EC
494.3
RTE1 LA
51.3
RTE1 Pca
1343.5
RTE-1 AC 1
602.2
RTE1X SP
613.0
RTE-2 ME
640.6
AviRTE MUn
50.7
AviRTE TiGu
39.7
AviRTE LLo
1586.5
RTE-3 OL
1287.4
EXPANDER2
188.9
Expander
202.5
EXPANDER1 DR
149.8
RTE-1 OL
1884.0
RTE1 ZM
165.7
RTE-1 TD
200.7
RTE2 ZM
641.5
RTE-1 ATr
612.5
RTE-1 STu
530.6
RTE-1 Mad
385.8
RTE-1 Alp
277.1
RTE1 MT
125.0
RTE-1 GM
outgroup
1500
1000
500
0
(MYA)
