# Supplementary material for: Long-Standing Activity with Characteristic Genomic Insertion Signatures in Reptilian Bov-B LINEs and Associated Sauria SINEs
Source: Biology (Basel). 2026 Jun 13;15(12):927. doi: 10.3390/biology15120927 (PMC13295347; doi:10.3390/biology15120927)
Supplement: Supplementary file 1 [file biology-15-00927-s001.zip › Supplementary_Material.pdf]

Legends for Supplementary Materials

Supplementary Table S1

Genome sequences and LINE/SINE sequences used in this study.

Supplementary Table S2

Query sequences used for the initial BLAST search and their internal direct repeats.

Supplementary Table S3

Genome survey for the Bov-B and Sauria SINE with the initial query sequences.

Supplementary Table S4

Query sequences used for the second BLAST search.

Supplementary Table S5

Copy numbers of the Bov-B and the number of direct repeats detected at different intervals.

Supplementary Table S6

The number of direct repeats detected beside Sauria SINEs at different intervals.

Supplementary Table S7

Discovered motif sites and statistical significance of motifs.

Supplementary Table S8

Comparison of Tn-TA trends and the first nucleotides of TSDs between LINEs and SINEs.

Supplementary Table S9

LINE sequences used in phylogenetic analysis.

Supplementary Figure S1

Python script for TSD search.

Supplementary Figure S2

Frequency distributions of sequence divergence and sequence length of LINE copies across snake and ruminant species.

The panels on the left indicate the frequency distribution of sequence divergence, and the

panels on the right indicate the frequency distribution of sequence length. All hits resulting from the second BLAST search for each species are used in the graphs.

Supplementary Figure S3

Sequence comparisons among Bov-B from *Podarcis muralis* with a length > 3 kb.

Supplementary Figure S4

Frequency distributions of sequence divergence and sequence length in the two *Indotyphlops braminus* Bov-B groups.

(A) Bov-B LINEs from *I. braminus* comprise young copies with small divergence and old copies with large divergence. (B) Frequency distributions of the lengths of young (left: Subfamily I) and old (right: Subfamily II) Bov-B.

Supplementary Figure S5

Sequence comparisons among young *I. braminus* Bov-B copies and their TSDs.

Full-length sequences of the Bov-B LINE from *I. braminus* with a small divergence (indicated by filled circles in Figure 3) were aligned. The 5′ and 3′ direct repeats are indicated in red. The middle of the LINEs was omitted for visibility.

Supplementary Figure S6

TSD search results with an interval of ≥2999 bases for Bov-B from *P. muralis*.

Supplementary Figure S7

Comparisons of the LINE/SINE motifs among different TSD intervals.

(A) Comparisons of motifs between LINEs and SINEs from *P. vitticeps* and other species. (B) Comparisons of Sauria SINE motifs across squamate species. The minimum motif width was set to 15 (*A. carolinensis*, *M. moschiferus*, *V. komodoensis* and *I. braminus*) or 29 (other species).

Supplementary Figure S8

MEME results for Bov-B from *P. muralis*.

Supplementary Figure S9

MEME results for Sauria SINE from *P. muralis*.

Supplementary Figure S10

ML tree of RTE-clade LINEs and bootstrap consensus tree.

The phylogenetic tree was constructed using the Maximum Likelihood method with 500 replicates, using the amino acid sequences of the ORF2 proteins. (A) ML tree. (B) Bootstrap consensus tree. Mammalian L1 LINEs were used as an outgroup.

Supplementary Figure S11

Estimation of the divergence time with different calibration constraints.

Divergence time estimation was performed using the RelTime method, using (A) the monocot/eudicot divergence time of 140 MYA [62, 63] or (B) the Toxicoferea/Serpentes divergence time of 184.6 MYA [64] as calibration constraints.

Supplementary Table S1

Genomic sequences and LINE/SINE sequences used in this study.

	Species	Common name	Availability of Genome assembly in Ensembl v110(1)	File name	NCBI accession	LINE sequences for First query	LINE sequences for Phylogenetic analysis	SINE sequences for First query	MEME analysis for LINE	MEME analysis for SINE
Squamates	*Salvator merianae*	Argentine black and white tegu	HLtupMer3	Salvator_merianae.HLtupMer3.dna.toplevel.fa	GCA_003586115.1	n/a	This study	n/a	✓	n/a
	Podarcis muralis	Common wall lizard	PodMur_1.0	Podarcis_muralis.PodMur_1.0.dna.toplevel.fa	GCA_004329235.1	n/a	This study	n/a	✓	✓
	Pogona vitticeps	Central bearded dragon	pvi1.1	Pogona_vitticeps.pvi1.1.dna.toplevel.fa	GCA_900067755.1	n/a	This study	n/a	✓	✓
	Anolis carolinensis	Green anole	AnoCar2.0v2	Anolis_carolinensis.AnoCar2.0v2.dna.toplevel.fa	GCA_000090745.2	Repbase	Repbase	(3)	✓	[29]
	Varanus komodoensis	Komodo dragon	ASM479886v1	Varanus_komodoensis.ASM479886v1.dna.toplevel.fa	GCA_004798865.1	n/a	This study	n/a	✓	✓
	Indotyphlops braminus	Brahminy blindsnake	n/a	Blindsnake_haplomerger2.fa(2)	n/a	n/a	This study	n/a	✓	✓
	Naja naja	Indian cobra	Nana_v5	Naja_naja.Nana_v5.dna.toplevel.fa	GCA_009733165.1	n/a	This study	n/a	✓	✓
	Pseudonaja textilis	Eastern brown snake	EBS10Xv2-PRI	Pseudonaja_textilis.EBS10Xv2-PRI.dna.toplevel.fa	GCA_900518735.1	n/a	n/a	n/a	✓	✓
	Notechis scutatus	Mainland tiger snake	TS10Xv2-PRI	Notechis_scutatus.TS10Xv2-PRI.dna.toplevel.fa	GCA_900518725.1	n/a	n/a	n/a	✓	✓
	Laticauda laticaudata	Blue-lipped sea krait	latLat_1.0	Laticauda_laticaudata.latLat_1.0.dna.toplevel.fa	GCA_004320025.1	n/a	n/a	n/a	✓	✓
	Vipera ammodytes	Sand viper	n/a	n/a	GCA_047301725.1	n/a	Repbase	n/a	n/a	n/a
	Agkistrodon contortrix	Eastern copperhead	n/a	n/a	n/a	Repbase	Repbase	n/a	n/a	n/a
	Azemiops feae	Fea's viper	n/a	n/a	GCA_023970755.1	n/a	n/a	(3)	n/a	n/a
	Python molurus	Indian python	n/a	n/a	n/a	n/a	Repbase	n/a	n/a	n/a
Mammals	*Bos taurus*	Cow	ARS-UCD1.2	Bos_taurus.ARS-UCD1.2.dna.toplevel.fa	GCA_002263795.2	Repbase	Repbase	n/a	✓	n/a
	Bison bison bison	American bison	Bison_UMD1.0	Bison_bison_bison.Bison_UMD1.0.dna.toplevel.fa	GCA_000754665.1	n/a	n/a	n/a	✓	n/a
	Capra hircus	Goat	ARS1	Capra_hircus.ARS1.dna.toplevel.fa	GCA_001704415.1	n/a	n/a	n/a	✓	n/a
	Ovis aries	Sheep	Oar_rambouillet_v1.0	Ovis_aries_rambouillet.Oar_rambouillet_v1.0.dna.toplevel.fa	GCA_002742125.1	n/a	n/a	n/a	✓	n/a
	Moschus moschiferus	Siberian musk deer	MosMos_v2_BIUU_UCD	Moschus_moschiferus.MosMos_v2_BIUU_UCD.dna.toplevel.fa	GCA_004024705.2	n/a	This study	n/a	✓	n/a
	Cervus hanglu yarkandensis	Yarkand deer	CEY_v1	Cervus_hanglu_yarkandensis.CEY_v1.dna.toplevel.fa	GCA_010411085.1	n/a	n/a	n/a	✓	n/a
Plants	*Medicago truncatula*	Barrel medic	[29]	[29]	GCA_000219495.2	[29]	Repbase	[29]	[29]	[29]
	Glycine max	Soybean	[29]	[29]	GCA_000004515.1	[29]	Repbase	[29]	[29]	[29]

(1) https://jul2023.archive.ensembl.org/index.html

(2) Khedkar et al. [51]; Kambayashi C., Personal communication.

(3) Piskurek et al. [49].

Repbase: https://www.girinst.org/repbase/

This study: Supplementary Table S4.

✓: analyzed in this study.

n/a: Not available.

[29]: Nishiyama E, Ohshima K. Cross-kingdom commonality of a novel insertion signature of RTE-related short retro-posons. Genome Biol Evol. 2018 10(6):1471–1483.

[49]: Piskurek O, Austin CC, Okada N. Sauria SINEs: Novel short interspersed retroposable elements that are wide-spread in reptile genomes. J Mol Evol. 2006 62(5):630–644.

[51]: Khedkar G, Kambayashi C, Tabata H, Takemura I, Minei R, Ogura A, Kurabayashi A. The draft genome sequence of the Brahminy blindsnake Indotyphlops braminus. Sci Data. 2022 9(1):410.

Supplementary Table S2

Query sequences used for the initial BLAST search and their internal direct repeats.

Queries[(1)]	References	Species	Length (bases)	5´direct repeat[(3)]	3´direct repeat[(3)]	Interval (bases)[(3)]
Bov-B (BovB)	Repbase	*Bos taurus*	3847	1072–1085	2238–2251	1152
BovB_ACo	Repbase	*Agkistrodon contortrix*	3282	561–572	2595–2606	2022
RTE_BOV_B_AC_1	Repbase	*Anolis carolinensis*	3224	1304–1314	2546–2556	1231
Sauria_ACA-1-15[(2)]	Piskurek et al. [49]	*Anolis carolinensis*	333	n/a	n/a	n/a
Sauria_AFE	Piskurek et al. [49]	*Azemiops feae*	343	n/a	n/a	n/a

(1) Used as the query against ruminants (BovB), reptiles, except for *A. carolinensis* (BovB_ACo), *A. carolinensis* (RTE_BOV_B_AC_1), lizards (Sauria_ACA-1-15), and snakes (Sauria_AFE).

(2) Clone ACA-1-15: GenBank accession number: FJ158974.

(3) Internal direct repeats found in the query sequences.

n/a: Not applicable.

Supplementary Table S3

Genome survey for the Bov-B and Sauria SINE with the initial query sequences.

clade	family	species	NCBI accession	Bov-B(1)	Sauria SINE(2)
Testudines	Podocnemididae	*Podocnemis expansa*	GCA_045364815.1	0	0
	Emydidae	*Trachemys scripta elegans*	GCA_013100865.1	0	0
Crocodilia	Alligatoridae	*Alligator mississippiensis*	GCA_030867095.1	0	0
	Crocodylidae	*Crocodylus porosus*	GCA_001723895.1	0	0
	Gavialidae	*Gavialis gangeticus*	GCA_001723915.1	0	0
Aves	Phasianidae	*Gallus gallus*	GCA_016699485.1	0	0
Sphenodontia	Sphenodontidae	*Sphenodon punctatus*	GCA_003113815.1	0	0
Gekkota	Diplodactylidae	*Correlophus ciliatus*	GCA_040207535.1	7	0
	Eublepharidae	*Coleonyx elegans*	GCA_043090795.1	6	25
	Sphaerodactylidae	*Euleptes europaea*	GCA_029931775.1	0	0
	Sphaerodactylidae	*Sphaerodactylus townsendi*	GCA_021028975.2	1	0
	Phyllodactylidae	*Asaccus caudivolvulus*	GCA_042257475.1	2444	0
	Phyllodactylidae	*Thecadactylus rapicauda*	GCA_044008395.1	283	9
	Gekkonidae	*Gekko gecko*	GCA_029375565.1	18796	26375
	Gekkonidae	*Heteronotia binoei*	GCA_032191835.1	6817	0
	Gekkonidae	*Mediodactylus kotschyi*	GCA_965636955.1	2784	4735
	Gekkonidae	*Paroedura picta*	GCA_049243985.1	35480	0
Scincomorpha	Cordylidae	*Hemicordylus capensis*	GCA_027244095.1	2	0
	Scincidae	*Tiliqua scincoides*	GCA_035046505.1	544	55
	Scincidae	*Marisora unimarginata*	GCA_046118615.1	842	854
	Scincidae	*Spondylurus nitidus*	GCA_041380405.1	427	193
	Scincidae	*Lerista edwardsae*	GCA_029204185.1	6864	12898
	Scincidae	*Mesoscincus schwartzei*	GCA_046270105.1	162	142
	Scincidae	*Plestiodon gilberti rubricaudatus*	GCA_026170595.1	13117	2129
Lacertoidea	Amphisbaenidae	*Rhineura floridana*	GCA_030035675.1	2	0
	Lacertidae	*Darevskia valentini*	GCA_024498535.1	1702	9571
	Lacertidae	*Lacerta agilis*	GCA_009819535.1	14585	7137
	Lacertidae	*Podarcis cretensis*	GCA_951804945.1	3985	8309
	Lacertidae	*Podarcis muralis*	GCA_004329235.1	3710	9516
	Lacertidae	*Zootoca vivipara*	GCA_963506605.1	6866	26470
	Teiidae	*Salvator merianae*	GCA_003586115.2	2611	0
	Teiidae	*Tupinambis teguixin*	GCA_047748235.1	514	0

	Teiidae	*Aspidoscelis marmoratus*	GCA_014337955.1	328	0
	Teiidae	*Aspidoscelis tigris stejnegeri*	GCA_023333525.2	325	0
	Teiidae	*Holcosus undulatus*	GCA_046270765.1	349	167
	Gymnophthalmidae	*Bachia flavescens*	GCA_044758885.1	523	838
	Gymnophthalmidae	*Calyptommatus sinebrachiatus*	GCA_022412395.1	47	0
	Gymnophthalmidae	*Tretioscincus oriximinensis*	GCA_022410635.1	39	0
Anguimorpha	Shinisauridae	*Shinisaurus crocodilurus*	GCA_021292165.1	0	0
	Varanidae	*Varanus acanthurus*	GCA_050042745.1	26161	9
	Varanidae	*Varanus komodoensis*	GCA_004798865.1	27501	12
	Varanidae	*Varanus salvator macromaculatus*	GCA_023646645.1	47890	24
	Anniellidae	*Anniella stebbinsi*	GCA_051312545.1	0	0
	Anguidae	*Dopasia gracilis*	GCA_052054735.1	0	0
	Anguidae	*Ophisaurus attenuatus*	GCA_046270065.1	84	167
	Anguidae	*Elgaria multicarinata webbii*	GCA_023053635.2	36	0
	Helodermatidae	*Heloderma charlesbogerti*	GCA_026122225.1	18063	2623
Iguania	Agamidae	*Laudakia wui*	GCA_040285375.1	235	863
	Agamidae	*Phrynocephalus versicolor*	GCA_023846285.1	1626	2860
	Agamidae	*Pogona vitticeps*	GCA_047335585.1	19689	2972
	Chamaeleonidae	*Bradypodion pumilum*	GCA_035047305.1	5028	31363
	Chamaeleonidae	*Chamaeleo calyptratus*	GCA_043643385.1	1081	4192
	Chamaeleonidae	*Furcifer pardalis*	GCA_030440675.1	1901	12681
	Iguanidae	*Anolis carolinensis*	GCA_035594765.1	125	97045
	Iguanidae	*Cyclura pinguis*	GCA_030412105.1	0	0
	Corytophanidae	*Basiliscus vittatus*	GCA_046270045.1	248	8
	Crotaphytidae	*Gambalia wislizenii*	GCA_030847615.1	2	0
	Phrynosomatidae	*Sceloporus tristichus*	GCA_016801065.1	36	69
Scolecophidia	Typhlopidae	*Indotyphlos braminus*	n/a[(3)]	2580	435
Constrictores	Boidae	*Boa constrictor*	GCA_046270485.1	1480	972
	Boidae	*Corallus caninus*	GCA_046270885.1	1693	807
	Boidae	*Candoia aspera*	GCA_035149785.1	647	1646
	Boidae	*Charina bottae*	GCA_023362775.1	16008	23824
	Pythonidae	*Python bivittatus*	GCA_000186305.2	1014	1
	Pythonidae	*Morelia carinata*	GCA_047032895.1	1516	0
	Pythonidae	*Morelia viridis*	GCA_027559625.1	632	0
	Pythonidae	*Liasis olivaceus*	GCA_030867105.1	6580	0
	Pythonidae	*Simalia tracyae*	GCA_031001705.1	3322	0

Caenophidia	Viperidae	*Azemiops feae*	GCA_023970755.1	6860	1692
	Viperidae	*Bothrops jararaca*	GCA_018340635.1	1304	644
	Viperidae	*Crotalus adamanteus*	GCA_039797435.1	24312	1319
	Viperidae	*Crotalus horridus*	GCA_051820455.1	21465	1392
	Viperidae	*Crotalus pyrrhus*	GCA_000737285.1	543	502
	Viperidae	*Crotalus viridis*	GCA_003400415.2	20529	1249
	Viperidae	*Protobothrops flavoviridis*	GCA_003402635.1	2057	939
	Viperidae	*Protobothrops mucrosquamatus*	GCA_001527695.3	3018	858
	Viperidae	*Daboia siamensis*	GCA_024449315.1	17981	10797
	Viperidae	*Vipera berus*	GCA_964194415.1	48784	26672
	Viperidae	*Vipera latastei*	GCA_024294585.1	43978	26466
	Viperidae	*Vipera ursinii*	GCA_947247035.1	43646	21033
	Homalopsidae	*Cerberus rynchops*	GCA_032468195.1	792	563
	Homalopsidae	*Myanophis thanlyinensis*	GCA_017656035.1	674	504
	Colubridae	*Thamnophis elegans*	GCA_009769535.1	14585	2760
	Colubridae	*Thamnophis sirtalis fitchi*	GCA_029207655.1	14648	2713
	Colubridae	*Tropidonophis doriae*	GCA_026931265.2	586	501
	Colubridae	*Diadophis punctatus*	GCA_023053685.1	7512	2395
	Colubridae	*Imantodes cenchoa*	GCA_027560375.1	821	536
	Colubridae	*Thermophis baileyi*	GCA_003457575.1	7000	2970
	Colubridae	*Ahaetulla prasina*	GCA_028640845.1	20557	5867
	Colubridae	*Chrysopelea ornata*	GCA_019457695.2	586	503
	Colubridae	*Arizona elegans*	GCA_022577455.1	13165	3512
	Colubridae	*Pantherophis guttatus*	GCA_029531705.1	13192	3965
	Colubridae	*Pantherophis obsoletus*	GCA_012654085.1	11823	3893
	Colubridae	*Pituophis catenifer annectens*	GCA_029215685.1	12388	3770
	Colubridae	*Ptyas mucosa*	GCA_012654045.1	12746	3492
	Psammophiidae	*Malpolon monspessulanus*	GCA_964265115.1	35385	2452
	Psammophiidae	*Psammophis punctulatus*	GCA_043091005.1	4082	664
	Psammophiidae	*Psammophis sibilans*	GCA_044335465.1	4662	724
	Lamprophiidae	*Boaedon fuliginosus*	GCA_027560395.1	1051	580
	Lamprophiidae	*Bofa erlangeri*	GCA_046269965.1	1867	885
	Pseudoxyrhophiidae	*Pseudoxyrhopus heterurus*	GCA_032468235.1	2461	563
	Psammodynastidae	*Psammodynastes pulverulentus*	GCA_025802295.1	6366	1686
	Elapidae	*Bungarus multicinctus*	GCA_023653725.1	3853	1224
	Elapidae	*Emydocephalus ijimae*	GCA_004319985.1	691	518

Elapidae	*Hydrophis curtus*	GCA_037043045.1	3486	1022
Elapidae	*Hydrophis cyanocinctus*	GCA_019473425.1	3688	1037
Elapidae	*Laticauda colubrina*	GCA_015471245.1	3342	867
Elapidae	*Laticauda laticaudata*	GCA_004320025.1	738	528
Elapidae	*Naja naja*	GCA_009733165.1	3997	1471
Elapidae	*Notechis scutatus*	GCA_900518725.1	3435	1428
Elapidae	*Pseudonaja textilis*	GCA_049901715.1	3422	1132
Atractaspididae	*Atractaspis fallax*	GCA_046269645.1	1761	885
Atractaspididae	*Atractaspis microlepidota*	GCA_043110025.1	1872	702

(1) Number of hits with BovB_ACo as the query for the initial BLAST search.

(2) Number of hits with Sauria_ACA-1-15 (lizards) or Sauria_AFE (snakes and other species) as the query for the initial BLAST search.

(3) Khedkar et al. [51]; Kambayashi C., Personal communication.

The blastn search (BLAST+ 2.9.0) was performed against the whole-genome sequences with default settings.

Supplementary Table S4

Query sequences used for the second BLAST search.

Retroposons	Queries	Species	Length (bases)	References
LINE	Bov-B_SMe	*Salvator merianae*	3081	This study
	Bov-B_PMu	*Podarcis muralis*	3249	This study
	Bov-B_PVi	*Pogona vitticeps*	3209	This study
	RTE_BOV_B_AC_1(1)	*Anolis carolinensis*(2)	3224	Repbase
	Bov-B_VKo	*Varanus komodoensis*	3266	This study
	Bov-B_IBr	*Indotyphlops braminus*	3218	This study
	Bov-B_NNa	*Naja naja*	3176	This study
	Bov-B_PTe	*Pseudonaja textilis*	3094	This study
	Bov-B_NSc	*Notechis scutatus*	3212	This study
	Bov-B_LLa	*Laticauda laticaudata*	3166	This study
	Bov-B (BovB)(1)	*Bos taurus*(2)	3847	Repbase
	Bov-B_BBi	*Bison bison bison*	3823	This study
	Bov-B_CHi	*Capra hircus*	3884	This study
	Bov-B_OAr	*Ovis aries*	3848	This study
	Bov-B_MMo	*Moschus moschiferus*	3707	This study
	Bov-B_CHa	*Cervus hanglu yarkandensis*	3713	This study
SINE	Sauria_POM	*Podarcis muralis*	326	This study
	Sauria_PVi	*Pogona vitticeps*	326	This study
	Sauria_VKo	*Varanus komodoensis*	256	This study
	Sauria_IBr	*Indotyphlops braminus*	337	This study
	Sauria_NNa	*Naja naja*	359	This study
	Sauria_PTe	*Pseudonaja textilis*	353	This study
	Sauria_NSc	*Notechis scutatus*	353	This study
	Sauria_LLa	*Laticauda laticaudata*	340	This study

(1) Consensus sequence of the LINE.

(2) The BLAST search was performed only once.

Supplementary Table S5

Copy numbers of the Bov-B and the number of direct repeats detected at different intervals.

Species		Copy length / TSD interval						
		>3700 / ≥3699	>3100 / ≥3099	>3000 / ≥2999	>2000 / ≥1999	>1000 / ≥999	>100 / ≥99	All
Salvator merianae	Copies(1)			11	114	661	3260	3393
	Direct repeats(2)			1	51	334 (329)	n.d.	n.d.
	Ratio(3)			0.09	0.45	0.51	n.d.	n.d.
	Tn-TA(4)			n.d.	n.d.	−	n.d.	n.d.
Podarcis muralis	Copies			435	770	2557	46867	52041
	Direct repeats			357 (353)	678	1786	26761	n.d.
	Ratio			0.82	0.88	0.70	0.57	n.d.
	Tn-TA			+	n.d.	n.d.	n.d.	n.d.
Pogona vitticeps	Copies		143	164	690	5460	43315	45093
	Direct repeats		63 (62)	105 (104)	310 (301)	2627 (2000)	21083	n.d.
	Ratio		0.44	0.64	0.45	0.48	0.49	n.d.
	Tn-TA		+	+	+	+/−(5)	n.d.	n.d.
Anolis carolinensis	Copies			65	286	2070	14941	15740
	Direct repeats			41 (41)	175 (173)	n.d.	7742	n.d.
	Ratio			0.63	0.61	n.d.	0.52	n.d.
	Tn-TA			+(6)	−	n.d.	n.d.	n.d.
Varanus komodoensis	Copies			1076	2699	7455	29500	31077
	Direct repeats			516 (509)	1602	4529	19060	n.d.
	Ratio			0.48	0.59	0.61	0.65	n.d.
	Tn-TA			+	n.d.	n.d.	n.d.	n.d.
Indotyphlops braminus	Copies			159	237	374	730	968
	Direct repeats			125 (123)	183	318	744	n.d.
	Ratio			0.79	0.77	0.85	1.02	n.d.
	Tn-TA			+	n.d.	n.d.	n.d.	n.d.
Naja naja	Copies			9	42	222	2268	2370
	Direct repeats			3	21	124 (120)	1349	n.d.
	Ratio			0.33	0.50	0.56	0.59	n.d.
	Tn-TA			n.d.	n.d.	−	n.d.	n.d.
Bos taurus	Copies	207		8199	29717	115786	348149	365688
	Direct repeats	176 (153)		3588 (2000)	18938	73014	248632	n.d.
	Ratio	0.85		0.44	0.64	0.63	0.71	n.d.
	Tn-TA	+		−	n.d.	n.d.	n.d.	n.d.

(1) Number of Bov-Bs of indicated length.

(2) The number of direct repeats detected at the indicated intervals.

The number of direct repeats used for MEME analysis is shown in parentheses.

(3) Num. of Direct repeats/ Num. of Copies

(4) Datasets that showed a Tn-TA pattern are indicated by +, while they did not show by −. n.d.: Not determined.

(5) A Tn-TA-like pattern was observed; however, the E-value was not significant (Figure S7A).

(6) Motif was statistically significant; however, the E-value was not significantly low (Figure S7A, Table S7).

Supplementary Table S6

The number of direct repeats detected beside Sauria SINEs at different intervals.

Species		TSD interval			
		≥299	≥199	≥99	All
Podarcis muralis	Copies[1]	n.d.	n.d.	n.d.	77228
	Direct repeats[2]	36368 (2000)	39223 (2000)	41553 (2000)	n.d.
	Tn-TA[3]	+	+	+	n.d.
Pogona vitticeps	Copies	n.d.	n.d.	n.d.	10602
	Direct repeats	4921 (2000)	5457 (2000)	5881 (2000)	n.d.
	Tn-TA	+	+	+	n.d.
Varanus komodoensis	Copies	n.d.	n.d.	n.d.	845
	Direct repeats	329(325)	364(359)	383(378)	n.d.
	Tn-TA	+	+	+	n.d.
Indotyphlops braminus	Copies	n.d.	n.d.	n.d.	497
	Direct repeats	87 (86)	112 (108)	133 (129)	n.d.
	Tn-TA	+[4]	+[4]	+[4]	n.d.
Naja naja	Copies	n.d.	n.d.	n.d.	1041
	Direct repeats	148 (141)	205 (196)	249 (233)	n.d.
	Tn-TA	+[4]	+[4]	+[4]	n.d.
Pseudonaja textilis	Copies	n.d.	n.d.	n.d.	986
	Direct repeats	138 (131)	184 (176)	236 (227)	n.d.
	Tn-TA	+[4]	+[4]	−[5]	n.d.
Notechis scutatus	Copies	n.d.	n.d.	n.d.	408
	Direct repeats	48 (47)	70 (70)	87 (86)	n.d.
	Tn-TA	n.d.	n.d.	−	n.d.
Laticauda laticaudata	Copies	n.d.	n.d.	n.d.	448
	Direct repeats	57 (54)	73 (70)	94 (90)	n.d.
	Tn-TA	−	−	−	n.d.

(1) Number of SINEs of indicated length.

(2) The number of direct repeats detected at the indicated intervals.
The number of direct repeats used for MEME analysis is shown in parentheses.

(3) Datasets that showed a Tn-TA pattern are indicated by +, while those that did not show by −.

(4) Motif was statistically significant; however, the E-value was not significantly low (Figure S7B, Table S7).

(5) Motif was statistically significant; however, the E-value was not significantly low, and the pattern was not clear (Figure S7B).

n.d.: Not determined.

Supplementary Table S7

Discovered motif sites and statistical significance of motifs.

Retroposons	Species	Common name	Motif sites[1]	All sites[2]	E-value[3]
Bov-B LINE	*Podarcis muralis*	Common wall lizard	265	353	4.2e−538
	Pogona vitticeps	Central bearded dragon	37	62	7.2e−036
	Varanus komodoensis	Komodo dragon	313	509	3.6e−572
	Indotyphlops braminus	Brahminy blindsnake	108	123	8.5e−193
	Anolis carolinensis	Green anole	37	41	6.0e−005[4]
	Bos taurus	Cow	42	153	7.4e−012
	Moschus moschiferus	Siberian musk deer	27	105	8.2e+003[5]
Sauria SINE	*Podarcis muralis*	Common wall lizard	1029	2000	2.5e−531
	Pogona vitticeps	Central bearded dragon	1028	2000	4.6e−344
	Varanus komodoensis	Komodo dragon	229	378	8.3e−261
	Indotyphlops braminus	Brahminy blindsnake	39	129	1.9e−008[4]
	Naja naja	Indian cobra	58	233	8.0e−016[4]
	Pseudonaja textilis	Eastern brown snake	73	176[6]	9.5e−014[4]

(1) Number of sites contributing to motif construction.

(2) Number of analyzed TSD sites.

(3) Statistical significance of the motif.

(4) Motif was statistically significant; however, the E-value was not significantly low.

(5) E-values were not statistically significant.

(6) TSD interval ≥199.

Supplementary Table S8

Comparison of Tn-TA trends and the first nucleotides of TSDs between LINEs and SINEs.

	Name	References	Species	Interval	Tn-TA trend [1]	TSD[2]	3′Repeat
LINE	Bov-B_SMe	This study	*Salvator merianae*	≥999	− (329)	?	unidentified
	Bov-B_PMu	This study	*Podarcis muralis*	≥2999	+ (265/353)	A	$(CAA)_{2-6}$
	Bov-B_PVi	This study	*Pogona vitticeps*	≥3099	+ (37/62)	A	$(CAA)_3/(CA)_{4-8}$
	RTE_BOV_B_AC_1	Repbase	*Anolis carolinensis*	≥2999	+ (37/41)[3]	A [29]	$(GCA)_{2-4}$
	Bov-B_VKo	This study	*Varanus komodoensis*	≥2999	+ (313/509)	A	$(CAA)_{2-4}$
	Bov-B_IBr	This study	*Indotyphlops braminus*	≥2999	+ (108/123)	A	$(CAA)_{2-5}$
	Bov-B_NNa	This study	*Naja naja*	≥999	− (120)	?	$(CAA)_{1-5}$
	Bov-B_PTe	This study	*Pseudonaja textilis*	≥499	− (393)	?	$(CAA)_{2-5}$
	Bov-B_NSc	This study	*Notechis scutatus*	≥999	− (120)	?	$(CAA)_{1-5}$
	Bov-B_LLa	This study	*Laticauda laticaudata*	≥999	− (68)	?	$(CAA)_{3-5}$
	Bov-B (BovB)	Repbase	*Bos taurus*	≥3699	+ (42/153)	A [29]	$(CTGAA)_{3-5}/(CTGAT)_{3-6}$
	Bov-B_BBi	This study	*Bison bison bison*	≥3499	− (82)	?	$(CTGAA)_{1-3}$
	Bov-B_CHi	This study	*Capra hircus*	≥3499	− (487)	?	$(CTGAA)_{1-4}$
	Bov-B_OAr	This study	*Ovis aries*	≥3499	− (408)	?	$(CTGAA)_{1-6}$
	Bov-B_MMo	This study	*Moschus moschiferus*	≥3499	+/− (27/105)[4]	A	$(CTGAA)_{3-4}$
	Bov-B_CHa	This study	*Cervus hanglu yarkandensis*	≥3499	− (38)	?	$(CTGAA)_{3-4}$
SINE	Sauria_POM	Repbase; [49]	*Podarcis muralis*	≥99	+ (1029/2000)	T	$(ACCTTT)_{1-2}$
	Sauria_PVi	This study	*Pogona vitticeps*	≥99	+ (1028/2000)	T	$(ACCTTT)_{1-3}$
	Sauria_ACA	Repbase; [49]	*Anolis carolinensis*	≥99	+ [29]	T [29]	$(ACCTTT)_{2-4}$
	Sauria_VKo	This study	*Varanus komodoensis*	≥99	+ (229/378)	T	$(ACCTTT)_{1-3}$
	Sauria_IBr	This study	*Indotyphlops braminus*	≥99	+ (39/129)[5]	T	$(ACCTTT)_{1-2}$
	Sauria_NNa	This study	*Naja naja*	≥99	+ (58/233)[5]	T	$(ACCTTT)_{1-2}$
	Sauria_PTe	This study	*Pseudonaja textilis*	≥199	+ (73/176)[5]	T	$(ACCTTT)_{1-3}$
	Sauria_NSc	This study	*Notechis scutatus*	≥99	− (86)	?	$(ACCTTT)_2$
	Sauria_LLa	This study	*Laticauda laticaudata*	≥99	− (90)	?	$(ACCTTT)_{1-2}$

(1) Species that exhibited a Tn-TA pattern in TSDs are indicated by +; those that did not by −. The number of sites contributing to the construction of the motif (for +) and the number of analyzed TSD sites are indicated in parentheses. Borderline cases are indicated by +/−.

(2) First nucleotides of TSDs.

(3) Motif was statistically significant; however, the E-value was not significantly low (Figure S7A, Tables S5 and S7).

(4) A Tn-TA-like pattern was observed; however, the E-value was not significant (Figure S7A, Table S7).
(5) Motif was statistically significant; however, the E-value was not significantly low (Figure S7B, Tables S6, S7).

Supplementary Table S9

LINE sequences used in phylogenetic analysis.

Clade	LINE[1]	Species	Length (bases)	ORF[3] (aa)
RTE	Bov-B_IBr[2]	*Indotyphlops braminus*	3218	1047
	Bov-B	*Bos taurus*	3847	1048
	RTE_BOV_B_AC_1	*Anolis carolinensis*	3224	1010
	RTE-1_EC	*Equus caballus*	3208	784
	RTE1_LA	*Loxodonta africana*	3233	615
	RTE1_Pca	*Procavia capensis*	3259	837
	RTE-1_AC_1	*Anolis carolinensis*	3912	957
	RTE1X_SP	*Strongylocentrotus purpuratus*	3561	891
	RTE-2_ME	*Notamacropus eugenii*	3314	935
	AviRTE_MUn	*Melopsittacus undulatus*	3999	1088
	AviRTE_TiGu	*Tinamus guttatus*	3999	1079
	AviRTE_LLo	*Loa loa*	3998	1064
	RTE-3_OL	*Oryzias latipes*	3401	923
	EXPANDER2	*Takifugu rubripes*	3369	1058
	Expander	*Takifugu rubripes*	3362	1058
	EXPANDER1_DR	*Danio rerio*	3365	1064
	RTE-1_OL	*Oryzias latipes*	3355	1058
	RTE1_ZM	*Zea mays*	3020	838
	RTE-1_TD	*Triticum durum*	3306	1040
	RTE2_ZM	*Zea mays*	3503	1040
	RTE-1_ATr	*Amborella trichopoda*	2579	784
	RTE-1_STu	*Solanum tuberosum*	4095	991
	RTE-1_Mad	*Malus domestica*	8343	1052
	RTE-1_AIp	*Arachis ipaensis*	3181	1020
	RTE1_MT	*Medicago truncatula*	3155	725
	RTE-1_GM	*Glycine max*	3181	1010
L1	L1HS	*Homo sapiens*	6064	1275
	L1-BT	*Bos taurus*	8390	1272

(1) Consensus sequences of LINEs, for which nearly full-length sequences were available, were obtained from Repbase, except for *Indotyphlops braminus*.

(2) identified in this study.

(3) Includes the EN and RT domains.
